# Eutypellenoids A–C, New Pimarane Diterpenes from the Arctic Fungus *Eutypella* sp. D-1

**DOI:** 10.3390/md16080284

**Published:** 2018-08-16

**Authors:** Hao-Bing Yu, Xiao-Li Wang, Wei-Heng Xu, Yi-Xin Zhang, Yi-Sen Qian, Jian-Peng Zhang, Xiao-Ling Lu, Xiao-Yu Liu

**Affiliations:** 1 Department of Biochemistry and Molecular Biology, College of Basic Medical Sciences, and Marine Biopharmaceutical Institute, Second Military Medical University, Shanghai 200433, China; yuhaobing1986@126.com (H.-B.Y.); xlwang0417@163.com (X.-L.W.); smmu_zyx@163.com (Y.-X.Z.); zjp80072002@hotmail.com (J.-P.Z.); luxiaoling80@126.com (X.-L.L.); 2 Center for Marine Biotechnology and Biomedicine, Scripps Institution of Oceanography, University of California, San Diego, CA 92093, USA; 3 School of Pharmacy, Second Military Medical University, Shanghai 200433, China; xuweiheng7114@163.com; 4 School of Physical Sciences, University of California, Irvine, CA 92697, USA; yisenq1@uci.edu

**Keywords:** Arctic fungus, *Eutypella* sp., pimarane diterpene, bioactivity

## Abstract

Three new pimarane diterpenes, eutypellenoids A–C (**1**–**3**), together with a known compound, eutypenoid C (**4**), were isolated from the culture extract of *Eutypella* sp. D-1 derived from the Arctic region. Compounds **1**–**3** possessed an uncommon tetrahydrofuran-fused pimarane diterpene skeleton. The structures of all compounds were determined by detailed spectroscopic analysis, electronic circular dichroism (ECD) analysis, as well as a comparison with the literature data. Antibacterial, antifungal, and cytotoxic activities of these compounds were evaluated. Compound **2** displayed antibacterial activity against *Staphylococcus aureus* and *Escherichia coli* with MIC values of 8 and 8 μg/mL, respectively. Additionally, compound **2** showed antifungal activity against *Candida parapsilosis*, *Candida albicans*, *Candida glabrata*, and *Candida tropicalis* with MIC values of 8, 8, 16, and 32 μg/mL, respectively. Furthermore, compound **2** exhibited moderate cytotoxic activity against HCT-116 cell line with IC_50_ value of 3.7 μM.

## 1. Introduction

Marine-derived fungi continue to embody an important spring of novel chemical structures with potentially useful applications as drugs [1]. Secondary metabolites from fungi of polar regions, including the Arctic, Antarctic, and their subregions, are rarely studied because of the challenging living environment [2]. The *Eutypella* species, found not only from the Arctic, but also from tropical forests and marine sources [2,3], produces a rich source of varied secondary metabolites, including pimarane diterpenoids, sesquiterpenoids, *γ*-lactones, cytochalasin derivatives, cyclic dipeptides, and cytosporin-related compounds [4,5,6,7,8,9,10]. Among them, pimarane diterpenes have attracted considerable interest because of their unique chemical structures and varied biological activities, such as antitumor, antimicrobial, and immunosuppressive activities [10,11].

**Figure 1:**
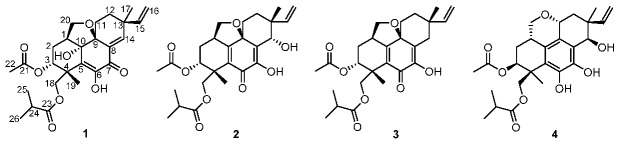
Chemical structures of compounds **1**–**4**.

As part of our continuous commitment to discovering structurally intriguing and biologically active natural products from polar fungi, we investigated the *Eutypella* sp. D-1, collected from the Arctic region, which led to the isolation of a series of cyclopropyl-fused and cyclobutyl-fused pimarane diterpenes with potent cytotoxic activity [9,10,11]. Subsequently, in an attempt to investigate the structurally diverse metabolites from this fungi, *Eutypella* sp. D-1 was further investigated by adding ethanol as an elicitor to the same liquid culture media as previously reported [11]. This work led to the isolation of three new pimarane diterpenes (**1**–**3**) with an uncommon tetrahydrofuran-fused skeleton (Figure 1).

## 2. Results

Eutypellenoid A (**1**) was obtained as a yellow oil and possessed a molecular formula of C_26_H_34_O_8_ by HRESIMS data (*m*/*z* 473.2187 [M − H]^−^), implying ten degrees of unsaturation. The UV spectrum exhibited absorptions at *λ*_max_ 202 and 301 nm. The IR absorptions at 3532, 3353, 1736, and 1659 cm^–1^ revealed the presence of hydroxyl, ester, and *α*,*β*-unsaturated carbonyl groups [9]. These spectroscopic characteristics and the initial inspections of the ^1^H and ^13^C NMR spectra indicated that **1** seemed to share common structural features with the libertellenone class [9]. The ^1^H NMR spectrum (Table 1) demonstrated signals assigned to five methyl groups at *δ*_H_ 1.13 (3H, d, 7.0 Hz), 1.14 (3H, d, 7.0 Hz), 1.23 (3H, s), 1.61 (3H, s), and 2.08 (3H, s), one olefinic proton at *δ*_H_ (7.11, s), and a terminal vinyl group at *δ*_H_ 5.71 (1H, dd, 17.5, 10.5 Hz), 5.04 (1H, d, 10.5 Hz), and 4.86 (1H, d, 17.5 Hz). Additionally, two hydroxyl protons were observed at *δ*_H_ 4.41 (s) and 7.17(s) from ^1^H NMR and HSQC spectra, respectively. The ^13^C NMR and DEPT spectra (Table 1) indicated the presence of 26 carbon resonances, including ten quaternary carbons (three carbonyl, three sp^2^, two sp^3^, and two oxygenated sp^3^ carbons), five methines (two sp^2^, one oxygenated sp^3^, and two sp^3^ carbons), six methylenes (one sp^2^, two oxygenated sp^3^, and three sp^3^ carbons), and five methyls. Apart from the three carbonyl groups (*δ*_C_ 181.3, 176.6, and 168.6) and three double bonds (*δ*_C_ 114.2, 124.7, 129.5, 142.7, 145.9, and 152.7), the remaining four degrees of unsaturation implied that **1** was likely to be a tetracyclic pimarane diterpenoid.

**Figure 2:**
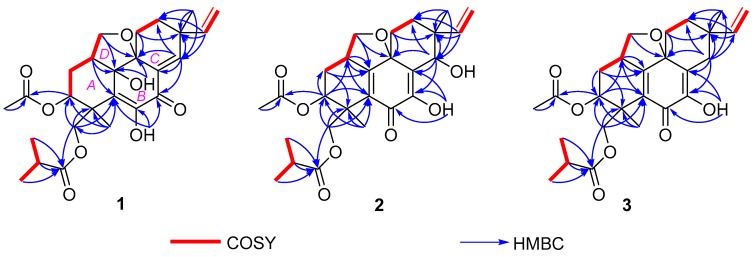
COSY and key HMBC correlations of compounds **1**–**3**.

The basic libertellenone carbon skeleton of **1** was confirmed by the COSY correlations from H-1/H-2*β*, H-2*β*/H-3, H-11*β*/H-12*β*, H-15/H_2_-16, and H_3_-25/H-24/H_3_-26, as well as the key HMBC correlations from H-1 to C-5 and C-10, from H-3 to C-21, from 6-OH to C-5, C-6, and C-7, from 10-OH to C-9 and C-10, from H-11β to C-9 and C-13, from H-14 to C-7, C-8, C-9, C-12, and C-13, from H-15 to C-12, C-13, and C-14, from H_3_-17 to C-12, C-13, and C-14, from H-18*α* to C-3, C-4, C-5, and C-23, from H_3_-19 to C-3, C-4, C-5, and C-18, from H_3_-22 to C-21, and from H-24, H_3_-25 and H_3_-26 to C-23 (Figure 2). Additionally, the remaining unsigned signals H-20*α* and H-20*β* showed COSY correlations with H-1 and HMBC correlations with C-1, C-2, C-9, and C-10 (Figure 2). These, taken together with the downfield shift of C-20 (*δ*_C_ 72.5) and the molecular formula, linked C-20 to C-9 and C-10 via an *O*-atom and C-1, respectively. Thus, eutypellenoid A (**1**) was elucidated as a new pimarane diterpene derivative.

**Figure 3:**
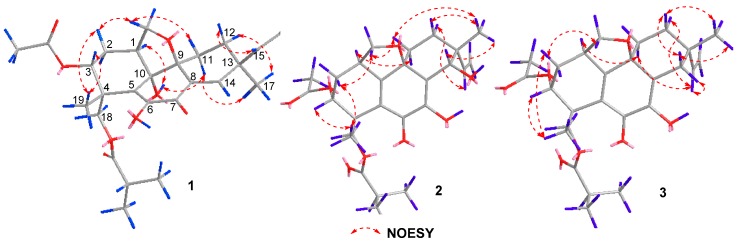
Key NOESY correlations of compounds **1**–**3**.

**Figure 4:**
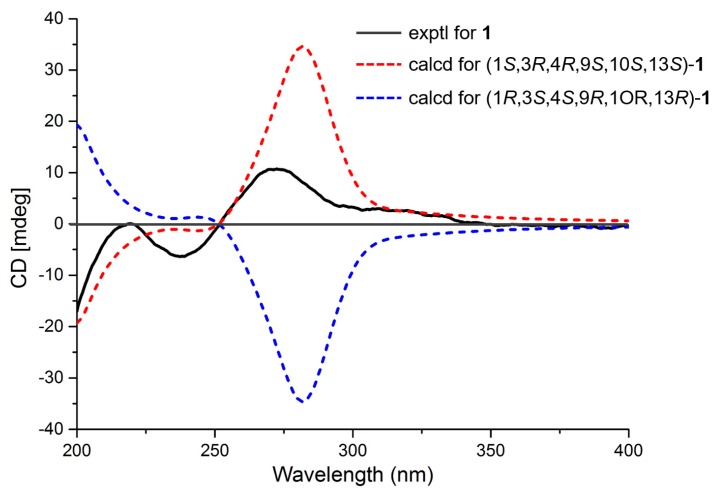
Experimental and calculated ECD spectra of compound **1**.

The relative configuration of compound **1** was established by a NOESY experiment (Figure 3). The NOESY correlations of H-1/10-OH, 10-OH/H-11*α*, H-11*α*/H_3_-17, and H-12*α*/H_3_-17 indicated that they were cofacial and assigned randomly as α-oriented. In addition, the NOESY correlations of H-2*β*/H_3_-19, H-2*β*/H-20*β*, H-3/H_3_-19, H-11*β*/H-20*β*, and H-15 /H-12*β* suggested the *β*-orientation for these protons (Figure 3). The absolute configuration of **1** was established by ECD experiments (Figure 4). The theoretical calculation of ECD was conducted in MeOH using time-dependent density functional theory (TD-DFT). The calculated ECD spectrum of 1*S*,3*R*,4*R*,9*S*,10*S*,13*S* was well matched with the experimental spectrum of **1**, thus determining the absolute configuration of **1** as 1*S*,3*R*,4*R*,9*S*,10*S*,13*S*.

**Figure 5:**
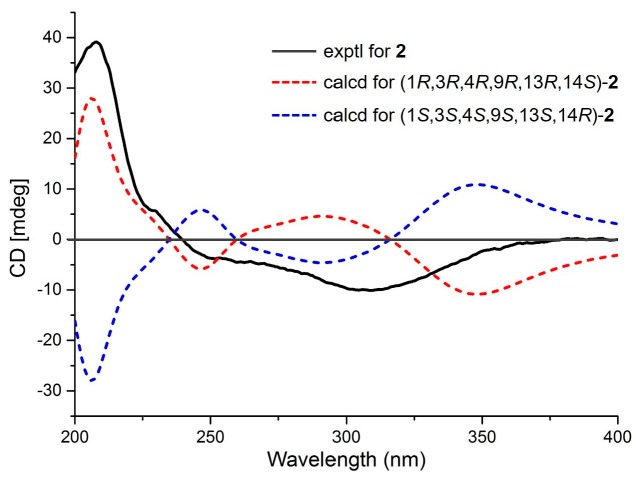
Experimental and calculated ECD spectra of compound **2**.

Eutypellenoid B (**2**) was also isolated as a yellow oil, with a the molecular formula C_26_H_34_O_8_ with 10 degrees of unsaturation based on HRESIMS data (*m*/*z* 492.2590 [M + NH_4_]^+^). The UV spectrum showed absorptions at *λ*_max_ 211 and 250 nm. The IR absorptions exhibited the presence of hydroxyl (3353 cm^−1^) and carbonyl carbons (1734 and 1640 cm^−1^) [9]. The ^1^H NMR spectrum (Table 1) also showed the characteristic pattern for a vinyl group at *δ*_H_ 5.72 (1H, dd, 17.6, 10.8 Hz), 5.09 (1H, d, 17.6 Hz), and 5.03 (1H, d, 10.8 Hz). A comparison of the ^1^H and ^13^C NMR data of **2** with those of compound **1** showed that they shared the same tetrahydrofuran-fused pimarane diterpene skeleton, except for the replacement of sp^2^ methine with a hydroxyl group at C-14 (*δ*_C_ 72.2), and the presence of a benzoquinone subunit in ring B of **2**. These was confirmed by the HMBC correlations (Figure 2) from H-3 to C-1, C-2, and C-5, from 7-OH to C-6, C-7, and C-8, from H-14 to C-7, C-8, C-9, C-12, and C-13, from H_3_-19 to C-3, C-4, and C-5, from H-20*β* to C-1, C-9, and C-10. The NOESY correlations of H-1/H-11*α*, H-12*α*/H-15, H-2*β*/H_3_-19, H-2*β*/H-20*β*, H-3/H_3_-19, H-11*β*/H_3_-17, H-11*β*/H-20*β*, H-12*β*/H_3_-17, and H-14/H_3_-17 established the relative configuration of **2** (Figure 3). Likewise, the experimental ECD spectrum of **2** was in good agreement with the calculated spectrum for 1*R*,3*R*,4*R*,9*R*,13*R*,14*S*, indicating that the absolute configuration of **2** is 1*R*,3*R*,4*R*,9*R*,13*R*,14*S* (Figure 5).

**Figure 6:**
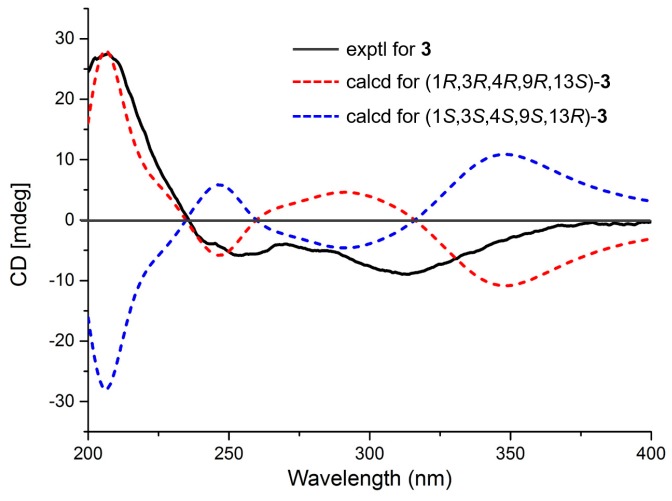
Experimental and calculated ECD spectra of compound **3**.

Eutypellenoid C (**3**) was isolated as a yellow oil. The molecular formula, C_26_H_34_O_7_, consistent with nine degrees of unsaturation, was determined based on HRESIMS data (*m*/*z* 457.2236 [M − H]^−^). The NMR spectra of **2** displayed close structure similarities to that of **3**, except for the absence of a hydroxyl group substituted at C-14 (*δ*_C_ 35.0). Therefore, C-8 was attached to C-13 via the methylene carbon C-14, which was supported by the HMBC correlations from H-14*α* and H-14*β* to C-7, C-8, C-9, C-12, C-13, C-15, and C-17. The NOESY cross-peaks (Figure 3) of H-1/H-11*α*, H-11*α*/H-15, H-12*α*/H-15, and H-14*α*/H-15 indicated that these protons were *α*-oriented, while the NOESY cross-peaks of H-2β/H_3_-19, H-2*β*/H-20*β*, H-3/H_3_-19, H-12β/H_3_-17, H-14β/H_3_-17, and H-12*β*/H-14*β* indicated that they were *β*-oriented. A comparison of the calculated and experimental ECD spectra of **3** determined its absolute configuration as 1*R*,3*R*,4*R*,9*R*,13*S* (Figure 6).

In addition to the three new compounds **1**−**3**, one known compound, eutypenoid C (**4**), was also obtained and elucidated by comparing the spectroscopic data with those reported in the literature [10].

All the isolated compounds were tested for antibacterial activity against *Staphylococcus aureus* (ATCC 27217), *Escherichia coli* (ATCC 25922), *Bacillus subtilis* (ATCC 21951), *Vibrio alginolyticus* (ATCC 33787), *Vibrio vulnificus* (ATCC 27562), *Streptococcus agalactiae* (ATCC 12386), and *Aeromonas hydrophila* (ATCC 35654) (Table 2), antifungal activity against *Candida parapsilosis* (ATCC 22019), *Candida albicans* (SC5314), *Candida glabrata* (537), *Cryptococcus neoformans* (32609), *Microsporum gypseum* (Cmccfmza), and *Candida tropicalis* (Table 3), and cytotoxic activities against HeLa (human cervical cancer cell line), MCF-7 (human breast adenocarcinoma cell line), HCT-116 (human colon carcinoma cancer cell line), K562 (human chronic myelogenous leukemia cell line), and SW1990 (human pancreatic cancer cell line) (Table 4). Compound **2** displayed antibacterial activity against *S. aureus* and *E. coli* with MIC values of 8 and 8 μg /mL, respectively. Compound **2** also showed antifungal activity against *C. parapsilosis*, *C. albicans*, *C. glabrata*, and *C. tropicalis* with MIC values of 8, 8, 16, and 32 μg/mL, respectively. Moreover, compound **2** exhibited moderate cytotoxic activity against HCT-116 cell line with IC_50_ value of 3.7 μM. The biological evaluation indicated that the hydroxylation at C-14, as in the case of comparison between **2** and **3**, correlated with a positive effect on the inhibitory activity. However, compared with the pimaranes with the cyclopropyl-fused or cyclobutyl-fused system [11], the tetrahydrofuran-fused system decreased the inhibitory activity.

## 3. Experimental Section

### 3.1. General Experimental Procedures

Optical rotations were measured on a Perkin-Elmer model 341 polarimeter (Perkin-Elmer Inc., Waltham, MA, USA). UV spectra were obtained on a UV-8000 spectrophotometer (Shanghai Metash instruments Co., Shanghai, China). IR (KBr) spectra of all compounds were performed on a Jasco FTIR-400 spectrometer (Jasco Inc., Tokyo, Japan). 1D and 2D NMR spectra were recorded on Bruker AMX-500 or Bruker AMX-400 instruments (Bruker Biospin Corp., Billerica, MA, USA) at room temperature (rt). HRESIMS data were obtained on an Agilent 6210 LC/MSD TOF mass spectrometer (Agilent Technologies Inc. Lake Forest, CA, USA). Semi-preparative HPLC chromatography was performed on a Waters 1525 separation module (Waters Corp., Milford, MA, USA) equipped with a Waters 2998 photodiode array (PDA) detector (Waters Corp., Milford, MA, USA) by using YMC-Pack Pro C_18_ RS (5 μm) columns (YMC Co. Ltd., Kyoto, Japan. Silica gel (200–300 mesh, Qingdao Ocean Chemical Co., Qingdao, China), ODS (50 μm, YMC Co. Ltd., Kyoto, Japan), and Sephadex LH-20 (18–110 μm, Pharmacia Co., Piscataway, NJ, USA) were used for column chromatography. 

### 3.2. Fungal Strain

The strain *Eutypella* sp. D-1 was isolated from the soil of London Island of Kongsfjorden of Ny-Ålesund District (altitude of 100 m) in the Arctic. It was incubated at 20 °C by using potato dextrose agar (PDA) medium. The fungus was identified as *Eutypella* sp. by 18S rDNA gene sequence analysis (GenBank accession number FJ430580). The strain was stored in PDA medium at the Second Military Medical University, Shanghai, China.

### 3.3. Fermentation, Extraction, and Isolation

The strain *Eutypella* sp. D-1 was maintained on PDA medium at 28 °C for seven days, and then three pieces (1 × 1 cm) of mycelial agar plugs were inoculated into 250 mL Erlenmeyer flasks, each containing 100 mL of seed medium (glucose 125 g/L, NaNO_3_ 3.3 g/L, MgSO_4_·7H_2_O 0.4 g/L, K_2_HPO_4_·3H_2_O 0.07 g/L, KCl 0.625 g/L, yeast extract 0.7 g/L, FeSO_4_·7H_2_O 18.75 mg/L, CoCl_2_·6H_2_O 3.125 mg/L, CaCl_2_ 6.5 mg/L, and _L_-ornithine hydrochloride 15 g/L, pH 5.8). After three days of incubation at 20 °C on a rotary shaker at 180 r/min, 20 mL seed cultures were transferred into a total of 150 flasks (2 L) containing 400 mL of fermentation medium (sucrose 51.4 g/L, NaNO_3_ 3.3 g/L, MgSO_4_·7H_2_O 0.4 g/L, K_2_HPO_4_·3H_2_O 0.07 g/L, KCl 0.625 g/L, yeast extract 0.7 g/L, FeSO_4_·7H_2_O 18.75 mg/L, CoCl_2_·6H_2_O 3.125 mg/L, and CaCl_2_ 6.5 mg/L, pH 5.8). The liquid cultivation was incubated for 10 days at 20 °C and 180 rpm on a rotary shaker. Meanwhile, 5 mL ethanol was added to the liquid cultivation three times, at 72 h, 96 h, and 120 h, respectively.

The whole culture (30 L) was filtered to give the broth and mycelia. The former was extracted with EtOAc three times, while the latter was extracted with a mixture of CH_2_Cl_2_/CH_3_OH (1:1, *v*/*v*) three times. The CH_2_Cl_2_/CH_3_OH solution was combined and evaporated under reduced pressure to obtain an aqueous solution and then extracted with EtOAc three times. These two EtOAc layers were almost the same by TLC and HPLC analysis, so they were combined and evaporated under reduced pressure to yield a dark brown gum (20.0 g).

The crude extract was subjected to vacuum liquid chromatography (VLC) on silica gel eluting with a step gradient of a mixture of petroleum ether (PE) and EtOAc (from 60:1 to 0:1) to afford seven fractions (A−G). Fraction C was separated on an ODS (50 μm) column followed by stepwise gradient elution with MeOH/H_2_O (3:5, 2:3, 4:5, 1:0) to obtain six subfractions (C1−C6). Fraction C4 was then further purified by HPLC (55% CH_3_CN/H_2_O, 2.0 mL/min) detected at the wavelength of 300 nm to yield compound **1** (t_R_ = 48.3 min, 2.0 mg) and **4** (t_R_ = 59.1 min, 3.8 mg). Fraction D was separated by CC on Sephadex LH-20 eluting with CH_2_Cl_2_/MeOH (1:1) to obtain three subfractions (D1−D3), and then fraction D2 was isolated by HPLC with an elution of 60% CH_3_CN detected at the wavelength of 252 nm to afford compound **2** (t_R_ = 37.6 min, 2.0 mg). Fraction E was separated on an ODS (50 μm) column followed by stepwise gradient elution with MeOH/H_2_O (50%→100%) to obtain five subfractions (E1−E5). Fraction E3 was then further purified by HPLC (65% CH_3_CN/H_2_O, 2.0 mL/min) to yield compound **3** (252 nm, t_R_ = 49.7 min, 2.0 mg).

Eutypellenoid A (**1**): yellow oil, [*α*]${}_{D}^{25}$ −12.0 (*c* 0.1, MeOH); UV (MeOH) *λ*_max_ (log *ε*) 202 (3.63), 301 (3.34) nm; IR (KBr) *v*_max_ 3532, 3353, 2962, 2928, 2874, 1736, 1659, 1620, 1464, 1371, 1342, 1224, 1159, 1076, 1034, 997, 955, 920 cm^−1^; CD (MeOH) (Δ*ε*) 219 (+0.1), 237 (−4.6), 272 (+7.6); ^1^H and ^13^C NMR data, see Table 1; HRESIMS *m*/*z* 473.2187 [M − H]^−^ (calcd for C_26_H_33_O_8_, 473.2193, Δ +1.24 ppm).

Eutypellenoid B (**2**): yellow oil, [*α*]${}_{D}^{25}$ −105.0 (*c* 0.1, MeOH); UV (MeOH) *λ*_max_ (log *ε*) 211 (3.62), 250 (3.23) nm; IR (KBr) *v*_max_ 3353, 2972, 2928, 1734, 1640, 1463, 1370, 1298, 1242, 1200, 1159, 1081, 1031, 987, 947, 921 cm^−1^; CD (MeOH) (Δε) 208 (+28.1), 229 (−7.2); ^1^H and ^13^C NMR data, see Table 1; HRESIMS *m*/*z* 492.2590 [M + NH_4_]^+^ (calcd for C_26_H_38_NO_8_, 492.2592, Δ +0.40 ppm).

Eutypellenoid C (**3**): yellow oil, [*α*]${}_{D}^{25}$ −100.0 (*c* 0.1, MeOH); UV (MeOH) *λ*_max_ (log *ε*) 208 (3.99), 249 (3.73), 320 (3.36) nm; IR (KBr) *v*_max_ 3404, 2961, 2929, 2879, 1736, 1658, 1629, 1464, 1371, 1346, 1299, 1241, 1195, 1156, 1076, 1032, 990, 948, 924, 890, 844, 803, 754 cm^−1^; CD (MeOH) (Δε) 207 (+19.1), 313 (−6.2); ^1^H and ^13^C NMR data, see Table 1; HRESIMS 457.2236 [M − H]^−^ (calcd for C_26_H_33_O_7_, 457.2232, Δ −0.99 ppm).

### 3.4. ECD Calculations

Conformational searches for compounds **1**–**3** were carried out via Spartan’s 10 software (Wave-function, Inc., Irvine, CA, USA) in the MMFF94 force field. Subsequently, the conformers with a Boltzmann population of over 5% were re-optimized at the B3LYP/6-31+G(d,p) level by employing the conductor-like polarizable continuum model (CPCM) in MeOH. The theoretical calculation of ECD for **1**–**3** were calculated using the time-dependent density functional theory (TDDFT) methodology at the B3LYP/6-311++G (2d, 2p) level in MeOH, respectively. The ECD spectra were generated by the program SpecDis 1.6 using a Gaussian function (*σ* = 0.3 eV, half the bandwidth at 1/e peak height) [12].

### 3.5. Biological Assays

The antimicrobial activities of compounds **1**–**4** against *E. coli*, *S. aureus*, *B. subtilis*, *V. vulnificus*, *V. alginolyticus*, *A. hydrophila*, and *S. agalactiae* were evaluated by the broth dilution method [13,14], and chloromycetin was used as a positive control. The antifungal activities of compounds **1**–**4** against *C. parapsilosis*, *C. albicans*, *C. glabrata*, *C. neoformans*, *M. gypseum*, and *C. tropicalis* were determined using the National Center for Clinical Laboratory Standards (NCCLS) methods [15,16,17]. Fluconazole, posaconazole, and voriconazole were used as the positive control. The cytotoxic activity of compounds **1**–**4** against HeLa, MCF-7, HCT-116, K562, and SW1990 cell lines was performed by the Cell Counting Kit-8 (CCK-8) assay, as described before [11,13]. Each cancer cell line was treated with the indicated test compound at various concentrations, in triplicate, and cisplatin was used as a positive control.

## 4. Conclusions

Investigation on the secondary metabolites from fungus *Eutypella* sp. D-1 isolated from the Arctic led to the isolation and structure elucidation of three new pimarane diterpenes (**1**–**3**), together with one known compound **4**. Structurally, compounds **1**–**3** possess an uncommon tetrahydrofuran-fused pimarane diterpene skeleton. These compounds were evaluated in antibacterial, antifungal, and cytotoxic activities. Only compound **2** displayed weak antibacterial activity against *S. aureus* and *E. coli* with MIC values of 8 and 8 μg/mL, respectively. Additionally, compound **2** showed antifungal activity against *C. parapsilosis*, *C. albicans*, *C. glabrata*, and *C. tropicalis* with MIC values of 8, 8, 16, and 32 μg/mL, respectively. Moreover, compound **2** exhibited moderate cytotoxic activity against HCT-116 cell line with IC_50_ value of 3.7 μM.

## Figures and Tables

**Table 1:** ^1^H and ^13^C NMR data of compounds **1**−**3** in CDCl_3_.

Position	1 ^a^		2 ^b^		3 ^b^	
	*δ*_C_	*δ*_H_, mult. (*J* in Hz)	*δ*_C_	*δ*_H_, mult. (*J* in Hz)	*δ*_C_	*δ*_H_, mult. (*J* in Hz)
1	40.6, CH	2.93, m	32.3, CH	3.27, m	33.0, CH	3.32, m
2α	26.0, CH_2_	2.32, m	26.5, CH_2_	2.35, m	26.8, CH_2_	2.33, m
2β		1.70, m		1.46, m		1.46, m
3	74.1, CH	5.11, t (7.0, 3.5)	72.7, CH	5.12, d (1.2)	72.9, CH	5.11, d (0.8)
4	42.8, C		40.5, C		40.5, C	
5	124.7, C		127.3, C		126.9, C	
6	145.9, C		180.3, C		179.9, C	
6-OH		7.17, s				
7	181.3, C		142.3, C		142.4, C	
7-OH				6.66, s		6.52, s
8	129.5, C		122.7, C		125.8, C	
9	80.9, C		79.2, C		77.7, C	
10	76.2, C		164.8, C		164.8, C	
10-OH		4.41, s				
11α	24.4, CH_2_	1.72, m	35.8, CH_2_	2.04, m	35.4, CH_2_	2.04, m
11β		2.09, m		1.54, m		1.41, m
12α	29.7, CH_2_	1.82, m	26.9, CH_2_	1.57, m	32.6, CH_2_	1.60, m
12β		1.64, m		2.08, m		1.93, m
13	39.8, C		44.9, C		41.1, C	
14α	152.7, CH	7.11, s	72.2, CH	4.68, s	35.0, CH_2_	2.96, dd (12.8, 2.0)
14β						2.15, d (12.8)
15	142.7, CH	5.71, dd (17.5, 10.5)	142.4, CH	5.72, dd (17.6, 10.8)	144.0, CH	5.68, dd (17.6, 10.8)
16a	114.2, CH_2_	4.86, d (17.5)	113.7, CH_2_	5.03, d (10.8)	112.9, CH_2_	4.96, d (10.8)
16b		5.04, d (10.5)		5.09, d (17.6)		5.12, d (17.6)
17	27.4, CH_3_	1.23, s	25.2, CH_3_	1.20, s	30.1, CH_3_	1.15, s
18*α*	64.9, CH_2_	4.42, d (14.0)	65.2, CH_2_	4.35, d (10.8)	65.3, CH_2_	4.35, d, (10.4)
18*β*		4.57, d (14.0)		4.79, d (10.8)		4.81, d (10.4)
19	20.6, CH_3_	1.61, s	20.9, CH_3_	1.31, s	20.9, CH_3_	1.31, s
20α	72.5, CH_2_	4.22, t (18.5, 9.5)	72.9, CH_2_	4.42, t (8.8)	71.9, CH_2_	4.36, t (8.0)
20β		3.47, t (18.0, 9.0)		3.77, t (8.8)		3.70, t (8.0)
21	168.6, C		169.9, C		170.0, C	
22	21.1, CH_3_	2.08, s	21.0, CH_3_	1.99, s	21.0, CH_3_	1.99, s
23	176.6, C		176.4, C		176.4, C	
24	34.1, CH	2.51, m	34.1, CH	2.52, m	34.1, CH	2.52, m
25	18.8, CH_3_	1.13, d (7.0)	18.8, CH_3_	1.13, d (6.8)	18.8, CH_3_	1.13, d (6.8)
26	18.9, CH_3_	1.14, d (7.0)	18.9, CH_3_	1.14, d (6.8)	18.9, CH_3_	1.13, d (6.8)

^a^ 500 MHz for ^1^H NMR and 125 MHz for ^13^C NMR. ^b^ 400 MHz for ^1^H NMR and 100 MHz for ^13^C NMR.

**Table 2:** Antibacterial activities of compounds **1**–**4**.

Compound	MIC (μg/mL)						
	*S. aureus*	*E. coli*	*B. subtilis*	*V. alginolyticus*	*V. vulnificus*	*S. agalactiae*	*A. hydrophila*
1	32	32	64	64	32	64	64
2	8	8	32	32	32	32	32
3	32	32	32	64	64	64	64
4	32	64	64	64	64	64	64
Chloromycetin	4	4	2	1	1	1	1

**Table 3:** Antifungal activities of compounds **1**–**4**.

Compound	MIC (μg/mL)					
	*C. parapsilosis*	*C. albicans*	*C. glabrata*	*C. neoformans*	*M. gypseum*	*C. tropicalis*
1	>64	>64	>64	>64	>64	>64
2	8	8	16	>64	>64	32
3	>64	>64	64	>64	>64	>64
4	>64	>64	64	>64	>64	>64
Fluconazole	0.50	2	0.50	1	2	0.25
Posaconazole	0.50	0.12	0.50	0.02	1	0.02
Voriconazole	0.02	0.03	0.02	0.02	0.06	0.02

**Table 4:** Cytotoxic activities of compounds **1**–**4**.

Compound	IC_50_ (μM)				
	HeLa	MCF-7	HCT-116	K562	SW1990
1	24.4	26.2	20.7	30.9	23.6
2	15.1	20.3	3.7	23.3	33.6
3	41.5	36.5	31.6	>50	>50
4	46.5	40.1	27.1	32.1	>50
Cisplatin	0.5	4.5	2.7	2.7	1.0

## Supplementary Materials

1D and 2D NMR, UV, IR, HRESMS data, and detailed ECD calculations of **1**–**3** are available online at http://www.mdpi.com/1660-3397/16/8/284/s1.
