# Supplementary material for: Eutypellenoids A–C, New Pimarane Diterpenes from the Arctic Fungus Eutypella sp. D-1
Source: Mar Drugs. 2018 Aug 16;16(8):284. doi: 10.3390/md16080284 (PMC6117666; doi:10.3390/md16080284)

Supplementary data

Eutypellenoids A–C, New Pimarane Diterpenes from the Arctic Fungus *Eutypella* sp. D-1

Hao-Bing Yu [1,2,†], Xiao-Li Wang [1,†], Wei-Heng Xu [3,†], Yi-Xin Zhang [1], Yi-Sen Qian [4], Jian-Peng Zhang [1], Xiao-Ling Lu [1] and Xiao-Yu Liu [1,*]

[1] Department of Biochemistry and Molecular Biology, College of Basic Medical Sciences, and Marine Biopharmaceutical Institute, Second Military Medical University, Shanghai 200433, China; yuhaobing1986@126.com (H.-B.Y.); xlwang0417@163.com (X.-L.W.); smmu_zyx@163.com (Y.-X.Z.); zjp80072002@hotmail.com (J.-P.Z.); luxiaoling80@126.com (X.-L.L.)
[2] Center for Marine Biotechnology and Biomedicine, Scripps Institution of Oceanography, University of California, San Diego, CA 92093, USA
[3] School of Pharmacy, Second Military Medical University, Shanghai 200433, China; xuweiheng7114@163.com
[4] School of Physical Sciences, University of California, Irvine, CA 92697, USA; yisenq1@uci.edu
* Correspondence: biolxy@163.com; Tel.: +86-21-6533-4344
† These authors contributed equally to this work.

S1 Quantum chemical CD calculation of eutypellenoid A (**1**).
S2 Quantum chemical CD calculation of eutypellenoid B (**2**).
S3 Quantum chemical CD calculation of eutypellenone C (**3**).
S4 ^{1}H NMR Spectrum of eutypellenoid A (**1**) in $CDCl_3$.
S5 ^{13}C NMR Spectrum of eutypellenoid A (**1**) in $CDCl_3$.
S6 DEPT135 Spectrum of eutypellenoid A (**1**) in $CDCl_3$.
S7 HSQC Spectrum of eutypellenoid A (**1**) in $CDCl_3$.
S8 COSY Spectrum of eutypellenoid A (**1**) in $CDCl_3$.
S9 HMBC Spectrum of eutypellenoid A (**1**) in $CDCl_3$.
S10 NOESY Spectrum of eutypellenoid A (**1**) in $CDCl_3$.
S11 HRESIMS of eutypellenoid A (**1**).
S12 UV spectrum of eutypellenoid A (**1**).
S13 IR spectrum of eutypellenoid A (**1**).
S14 ^{1}H NMR Spectrum of eutypellenoid B (**2**) in $CDCl_3$.
S15 ^{13}C NMR Spectrum of eutypellenoid B (**2**) in $CDCl_3$.
S16 DEPT135 Spectrum of eutypellenoid B (**2**) in $CDCl_3$.
S17 HSQC Spectrum of eutypellenoid B (**2**) in $CDCl_3$.
S18 COSY Spectrum of eutypellenoid B (**2**) in $CDCl_3$.
S19 HMBC Spectrum of eutypellenoid B (**2**) in $CDCl_3$.
S20 NOESY Spectrum of eutypellenoid B (**2**) in $CDCl_3$.
S21 HRESIMS of eutypellenoid B (**2**).
S22 UV spectrum of eutypellenoid B (**2**).
S23 IR spectrum of eutypellenoid B (**2**).
S24 ^{1}H NMR Spectrum of eutypellenoid C (**3**) in $CDCl_3$.
S25 ^{13}C NMR Spectrum of eutypellenoid C (**3**) in $CDCl_3$.
S26 DEPT135 Spectrum of eutypellenoid C (**3**) in $CDCl_3$.
S27 HSQC Spectrum of eutypellenoid C (**3**) in $CDCl_3$.
S28 COSY Spectrum of eutypellenoid C (**3**) in $CDCl_3$.
S29 HMBC Spectrum of eutypellenoid C (**3**) in $CDCl_3$.
S30 NOESY Spectrum of eutypellenoid C (**3**) in $CDCl_3$.
S31 HRESIMS of eutypellenoid C (**3**).
S32 UV spectrum of eutypellenoid C (**3**).
S33 IR spectrum of eutypellenoid C (**3**).

S1 Quantum chemical CD calculation of eutypellenoid A (**1**).

ECD calculation details

1. Methods

Monte Carlo conformational searches were carried out by means of the Spartan's 10 software using Merck Molecular Force Field (MMFF). The conformers with Boltzmann-population of over 5% were chosen for ECD calculations, and then the conformers were initially optimized at B3LYP/6-31+g (d, p) level in MeOH using the CPCM polarizable conductor calculation model. The theoretical calculation of ECD was conducted in MeOH using Time-dependent Density functional theory (TD-DFT) at the B3LYP/6-311+g (d, p) level for all conformers of compounds **1** Rotatory strengths for a total of 50 excited states were calculated. ECD spectra were generated using the program SpecDis 1.6 (University of Würzburg, Würzburg, Germany) and GraphPad Prism 5 (University of California San Diego, USA) from dipole-length rotational strengths by applying Gaussian band shapes with sigma = 0.3 eV.

2. Results

Table S1.2.1.Gibbs free energies[a] and equilibrium populations[b] of low-energy conformers of **1**

Conformers	In MeOH	
	ΔG	P (%)
**1a**	0.05	32.61
**1b**	0.35	19.88
**1c**	0	33.88
**1d**	0.67	12.68
**1e**	1.89	0.95

[a]B3LYP/6-31+G(d,p), in kcal/mol. [b]From ΔG values at 298.15K.

Table S1.2.2.Cartesian coordinates for the low-energy reoptimized MMFF conformers of **1** at B3LYP/6-311+G(d,p) level of theory in CH_3OH.

**1a**		Standard Orientation (Ångstroms)			
I	Atom	Type	X	Y	Z
1.	6.	0.	1.755662	-2.236006	-1.179906
2.	6.	0.	2.457115	-1.661734	0.039987
3.	6.	0.	2.081083	-0.188593	0.405273
4.	6.	0.	0.532767	-0.102258	0.464685
5.	6.	0.	-0.274132	-0.723478	-0.698402
6.	6.	0.	0.238331	-2.133200	-1.067915
7.	6.	0.	-0.139095	0.569894	1.435767
8.	6.	0.	-1.625589	0.653445	1.523254
9.	6.	0.	-2.448319	-0.069400	0.551284
10.	6.	0.	-1.761778	-1.077827	-0.336820
11.	6.	0.	-3.768487	0.202633	0.489677
12.	6.	0.	-4.743946	-0.405070	-0.478941
13.	6.	0.	-4.084638	-1.606908	-1.206076

14.	6.	0.	-2.627030	-1.330190	-1.588607
15.	6.	0.	2.798499	0.102066	1.746703
16.	6.	0.	2.654503	0.777410	-0.672345
17.	8.	0.	2.607185	2.162036	-0.224959
18.	6.	0.	1.649705	2.992918	-0.656471
19.	6.	0.	1.847924	4.411162	-0.151748
20.	6.	0.	0.586757	4.895924	0.584886
21.	6.	0.	2.201241	5.331636	-1.337309
22.	8.	0.	0.726418	2.656099	-1.390564
23.	8.	0.	3.893797	-1.718846	-0.191215
24.	6.	0.	4.575724	-2.802938	0.241423
25.	6.	0.	6.036239	-2.703088	-0.109871
26.	8.	0.	4.055767	-3.734333	0.831902
27.	6.	0.	-0.448865	-3.028643	-0.026703
28.	8.	0.	-1.616867	-2.303139	0.428970
29.	6.	0.	-5.147560	0.704106	-1.495710
30.	6.	0.	-6.011541	-0.881156	0.213485
31.	6.	0.	-6.242116	-0.970376	1.527329
32.	8.	0.	-2.086919	1.354426	2.438503
33.	8.	0.	0.445892	1.268255	2.441241
34.	8.	0.	-0.187042	0.096935	-1.861889
35.	1.	0.	2.054150	-3.282769	-1.303313
36.	1.	0.	2.084917	-1.704246	-2.078946
37.	1.	0.	2.244883	-2.274658	0.918516
38.	1.	0.	-0.172506	-2.357274	-2.055643
39.	1.	0.	-4.156033	0.963085	1.164835
40.	1.	0.	-4.118111	-2.481961	-0.548108
41.	1.	0.	-4.666841	-1.856316	-2.100046
42.	1.	0.	-2.237701	-2.187043	-2.144227
43.	1.	0.	-2.550890	-0.462451	-2.249932
44.	1.	0.	2.383176	-0.512484	2.549185
45.	1.	0.	2.720820	1.144561	2.040727
46.	1.	0.	3.857330	-0.145613	1.634495
47.	1.	0.	3.718540	0.579548	-0.797791
48.	1.	0.	2.149500	0.690599	-1.632119
49.	1.	0.	2.690115	4.394286	0.546027
50.	1.	0.	-0.273487	4.920213	-0.091049
51.	1.	0.	0.753039	5.907850	0.966562
52.	1.	0.	0.342521	4.247734	1.432259
53.	1.	0.	3.108837	4.998247	-1.850541
54.	1.	0.	1.382427	5.360633	-2.062864
55.	1.	0.	2.372313	6.348256	-0.970642
56.	1.	0.	6.578812	-3.538106	0.332313

57.	1.	0.	6.153230	-2.725371	-1.197974
58.	1.	0.	6.448610	-1.755313	0.246960
59.	1.	0.	0.179318	-3.226875	0.848413
60.	1.	0.	-0.759503	-3.989444	-0.451376
61.	1.	0.	-5.906689	0.316457	-2.183405
62.	1.	0.	-4.287619	1.036581	-2.083687
63.	1.	0.	-5.565634	1.573772	-0.979185
64.	1.	0.	-6.793411	-1.194381	-0.479874
65.	1.	0.	-7.191629	-1.343553	1.900283
66.	1.	0.	-5.505812	-0.685344	2.274016
67.	1.	0.	-0.306103	1.642499	2.950649
68.	1.	0.	0.008851	1.025435	-1.622004

**1b**		Standard Orientation (Ångstroms)			
I	Atom	Type	X	Y	Z
1.	6.	0.	1.517262	-2.366884	-1.137148
2.	6.	0.	2.286303	-1.792144	0.041364
3.	6.	0.	2.018992	-0.281492	0.345357
4.	6.	0.	0.481580	-0.098686	0.454144
5.	6.	0.	-0.400439	-0.710704	-0.658854
6.	6.	0.	0.013072	-2.162876	-0.989512
7.	6.	0.	-0.116931	0.634069	1.429519
8.	6.	0.	-1.591953	0.815806	1.557062
9.	6.	0.	-2.487114	0.121873	0.629967
10.	6.	0.	-1.894407	-0.959550	-0.238067
11.	6.	0.	-3.785410	0.487637	0.583890
12.	6.	0.	-4.841361	-0.109143	-0.317341
13.	6.	0.	-4.273225	-1.373324	-1.012977
14.	6.	0.	-2.815121	-1.201894	-1.451428
15.	6.	0.	2.805620	0.030590	1.642069
16.	6.	0.	2.611554	0.587505	-0.801961
17.	8.	0.	2.682815	1.993283	-0.433423
18.	6.	0.	1.778438	2.872871	-0.878225
19.	6.	0.	2.131650	4.305312	-0.516330
20.	6.	0.	3.362409	4.768476	-1.322597
21.	6.	0.	2.342947	4.487351	0.996996
22.	8.	0.	0.785568	2.566036	-1.531452
23.	8.	0.	3.709222	-1.960022	-0.217059
24.	6.	0.	4.328324	-3.061458	0.263657
25.	6.	0.	5.786213	-3.073394	-0.111628
26.	8.	0.	3.761236	-3.923925	0.912580

27.	6.	0.	-0.696992	-2.977464	0.101802
28.	8.	0.	-1.798778	-2.162535	0.569409
29.	6.	0.	-5.244525	0.965888	-1.353775
30.	6.	0.	-6.010722	-0.518952	0.570980
31.	6.	0.	-7.262210	-0.049688	0.536135
32.	8.	0.	-1.979949	1.569706	2.464271
33.	8.	0.	0.540403	1.315383	2.401098
34.	8.	0.	-0.304904	0.059066	-1.855513
35.	1.	0.	1.745918	-3.434514	-1.227202
36.	1.	0.	1.853459	-1.891423	-2.064702
37.	1.	0.	2.053088	-2.349458	0.951032
38.	1.	0.	-0.439607	-2.394184	-1.957107
39.	1.	0.	-4.107490	1.297446	1.235984
40.	1.	0.	-4.333820	-2.222805	-0.323058
41.	1.	0.	-4.899999	-1.621200	-1.876392
42.	1.	0.	-2.498459	-2.100390	-1.987352
43.	1.	0.	-2.709073	-0.364379	-2.146902
44.	1.	0.	2.395232	-0.524483	2.489036
45.	1.	0.	2.792665	1.087926	1.889658
46.	1.	0.	3.845252	-0.275768	1.499672
47.	1.	0.	3.652796	0.306043	-0.957290
48.	1.	0.	2.062152	0.487515	-1.735630
49.	1.	0.	1.265173	4.895785	-0.829266
50.	1.	0.	4.254111	4.202948	-1.035296
51.	1.	0.	3.549214	5.828069	-1.122677
52.	1.	0.	3.207093	4.649321	-2.399650
53.	1.	0.	1.470768	4.154860	1.568022
54.	1.	0.	3.215704	3.926396	1.343555
55.	1.	0.	2.508419	5.547302	1.213575
56.	1.	0.	6.271084	-3.946368	0.324180
57.	1.	0.	5.887285	-3.100507	-1.200897
58.	1.	0.	6.273022	-2.159940	0.241922
59.	1.	0.	-0.054491	-3.190244	0.963031
60.	1.	0.	-1.083721	-3.928174	-0.281271
61.	1.	0.	-6.053500	0.592892	-1.990045
62.	1.	0.	-4.396361	1.220583	-1.994596
63.	1.	0.	-5.584506	1.885421	-0.867313
64.	1.	0.	-5.763031	-1.288711	1.302729
65.	1.	0.	-8.014578	-0.432611	1.219780
66.	1.	0.	-7.585524	0.715962	-0.162452
67.	1.	0.	-0.170543	1.752247	2.919404
68.	1.	0.	-0.036365	0.980180	-1.660356

**1c**		Standard Orientation<br>(Ångstroms)			
I	Atom	Type	X	Y	Z
1.	6.	0.	1.646499	-2.282937	-1.182716
2.	6.	0.	2.402833	-1.732626	0.015293
3.	6.	0.	2.095740	-0.244160	0.382868
4.	6.	0.	0.554139	-0.097273	0.485636
5.	6.	0.	-0.307856	-0.689822	-0.653125
6.	6.	0.	0.138054	-2.120126	-1.029266
7.	6.	0.	-0.064575	0.598199	1.475482
8.	6.	0.	-1.544139	0.740602	1.602611
9.	6.	0.	-2.417657	0.058742	0.647328
10.	6.	0.	-1.797119	-0.983674	-0.248490
11.	6.	0.	-3.722789	0.399362	0.601041
12.	6.	0.	-4.749201	-0.181636	-0.339536
13.	6.	0.	-4.165207	-1.423041	-1.040750
14.	6.	0.	-2.708516	-1.215386	-1.471176
15.	6.	0.	2.862254	0.024955	1.701459
16.	6.	0.	2.675296	0.693375	-0.716350
17.	8.	0.	2.696056	2.080476	-0.275068
18.	6.	0.	1.758678	2.947212	-0.679144
19.	6.	0.	2.028349	4.357690	-0.185782
20.	6.	0.	0.814235	4.890122	0.595721
21.	6.	0.	2.372085	5.262733	-1.385801
22.	8.	0.	0.799894	2.645337	-1.382306
23.	8.	0.	3.829324	-1.848491	-0.253653
24.	6.	0.	4.480156	-2.953224	0.174496
25.	6.	0.	5.936929	-2.906635	-0.202438
26.	8.	0.	3.939058	-3.859858	0.784213
27.	6.	0.	-0.555141	-2.983201	0.035566
28.	8.	0.	-1.675913	-2.206964	0.525136
29.	6.	0.	-5.124040	0.924684	-1.372865
30.	6.	0.	-6.004676	-0.474728	0.474419
31.	6.	0.	-6.673417	-1.629609	0.548351
32.	8.	0.	-1.953275	1.455306	2.532149
33.	8.	0.	0.573517	1.271012	2.466065
34.	8.	0.	-0.220324	0.121925	-1.822408
35.	1.	0.	1.900709	-3.340885	-1.310379
36.	1.	0.	1.970863	-1.766836	-2.092628
37.	1.	0.	2.190144	-2.333417	0.902070
38.	1.	0.	-0.308699	-2.331436	-2.004170
39.	1.	0.	-4.064096	1.179850	1.279323
40.	1.	0.	-4.201818	-2.277709	-0.357115

41.	1.	0.	-4.778091	-1.681374	-1.911002
42.	1.	0.	-2.373433	-2.096912	-2.023473
43.	1.	0.	-2.613231	-0.362098	-2.148676
44.	1.	0.	2.447869	-0.569459	2.519404
45.	1.	0.	2.832394	1.070965	1.992047
46.	1.	0.	3.907582	-0.262362	1.560805
47.	1.	0.	3.726586	0.452955	-0.871550
48.	1.	0.	2.139310	0.622618	-1.660519
49.	1.	0.	2.894242	4.309795	0.480853
50.	1.	0.	-0.068268	4.948898	-0.048679
51.	1.	0.	1.033306	5.894343	0.971090
52.	1.	0.	0.575684	4.250828	1.451454
53.	1.	0.	3.246598	4.894293	-1.931521
54.	1.	0.	1.528526	5.322031	-2.080518
55.	1.	0.	2.595252	6.272537	-1.028388
56.	1.	0.	6.444548	-3.791318	0.180613
57.	1.	0.	6.036742	-2.865474	-1.291305
58.	1.	0.	6.401113	-2.003983	0.205408
59.	1.	0.	0.090381	-3.207220	0.891595
60.	1.	0.	-0.920054	-3.930287	-0.376688
61.	1.	0.	-5.905367	0.550133	-2.041803
62.	1.	0.	-4.257461	1.215548	-1.973279
63.	1.	0.	-5.502800	1.820046	-0.869742
64.	1.	0.	-6.389504	0.385937	1.023575
65.	1.	0.	-7.578582	-1.705787	1.144136
66.	1.	0.	-6.359629	-2.527617	0.025084
67.	1.	0.	-0.149856	1.672753	2.995654
68.	1.	0.	0.016865	1.043120	-1.591693

**1d**		Standard Orientation (Ångstroms)			
I	Atom	Type	X	Y	Z
1.	6.	0.	1.394352	-2.395173	-1.145315
2.	6.	0.	2.196971	-1.857790	0.028393
3.	6.	0.	1.993111	-0.339124	0.342326
4.	6.	0.	0.465206	-0.094621	0.463150
5.	6.	0.	-0.446478	-0.659041	-0.651964
6.	6.	0.	-0.098677	-2.127256	-0.986870
7.	6.	0.	-0.099076	0.643852	1.454473
8.	6.	0.	-1.564353	0.893067	1.585173
9.	6.	0.	-2.488304	0.264000	0.641318
10.	6.	0.	-1.948625	-0.842791	-0.228070
11.	6.	0.	-3.761985	0.706004	0.583794

12.	6.	0.	-4.831189	0.182520	-0.342755
13.	6.	0.	-4.350063	-1.125730	-0.998588
14.	6.	0.	-2.883181	-1.048734	-1.437997
15.	6.	0.	2.803298	-0.065657	1.633171
16.	6.	0.	2.612027	0.512587	-0.804238
17.	8.	0.	2.739920	1.912114	-0.426725
18.	6.	0.	1.873063	2.831024	-0.866348
19.	6.	0.	2.284516	4.245264	-0.494861
20.	6.	0.	3.530940	4.664367	-1.301343
21.	6.	0.	2.507021	4.407417	1.019053
22.	8.	0.	0.868849	2.569679	-1.522194
23.	8.	0.	3.609295	-2.081005	-0.245536
24.	6.	0.	4.190604	-3.205386	0.229224
25.	6.	0.	5.641853	-3.275526	-0.164994
26.	8.	0.	3.598315	-4.044275	0.886500
27.	6.	0.	-0.837964	-2.910004	0.108718
28.	8.	0.	-1.902009	-2.048236	0.580470
29.	6.	0.	-5.111682	1.280356	-1.414214
30.	6.	0.	-6.107090	0.021477	0.476130
31.	6.	0.	-6.869586	-1.070726	0.586712
32.	8.	0.	-1.917556	1.648250	2.505496
33.	8.	0.	0.588403	1.275528	2.438855
34.	8.	0.	-0.319581	0.109728	-1.845678
35.	1.	0.	1.576748	-3.471184	-1.241233
36.	1.	0.	1.743372	-1.930664	-2.073689
37.	1.	0.	1.950975	-2.410226	0.937657
38.	1.	0.	-0.566991	-2.337990	-1.951888
39.	1.	0.	-4.040609	1.526770	1.242848
40.	1.	0.	-4.452203	-1.949453	-0.284129
41.	1.	0.	-4.984632	-1.366115	-1.858392
42.	1.	0.	-2.620559	-1.973856	-1.957366
43.	1.	0.	-2.725495	-0.231640	-2.147408
44.	1.	0.	2.384273	-0.614409	2.479950
45.	1.	0.	2.828846	0.989277	1.890182
46.	1.	0.	3.830416	-0.405494	1.476099
47.	1.	0.	3.640615	0.191702	-0.967330
48.	1.	0.	2.053786	0.439968	-1.735082
49.	1.	0.	1.441828	4.872552	-0.801056
50.	1.	0.	4.399758	4.060897	-1.021147
51.	1.	0.	3.761086	5.713902	-1.093751
52.	1.	0.	3.367927	4.560114	-2.378791
53.	1.	0.	1.623160	4.107376	1.590128
54.	1.	0.	3.356719	3.808450	1.359288

55.	1.	0.	2.716570	5.458048	1.242791
56.	1.	0.	6.099010	-4.163204	0.271083
57.	1.	0.	5.726631	-3.314986	-1.255324
58.	1.	0.	6.168086	-2.378713	0.174246
59.	1.	0.	-0.201145	-3.148975	0.967245
60.	1.	0.	-1.266766	-3.843703	-0.271351
61.	1.	0.	-5.919012	0.948293	-2.074726
62.	1.	0.	-4.222393	1.479471	-2.018976
63.	1.	0.	-5.418126	2.219691	-0.943096
64.	1.	0.	-6.419302	0.928835	0.995582
65.	1.	0.	-7.777763	-1.051199	1.182499
66.	1.	0.	-6.632210	-2.009071	0.094800
67.	1.	0.	-0.101859	1.736263	2.964354
68.	1.	0.	-0.014093	1.018738	-1.647987

**1e**		Standard Orientation (Ångstroms)			
I	Atom	Type	X	Y	Z
1.	6.	0.	1.849927	-2.101000	-1.252299
2.	6.	0.	2.579727	-1.472854	-0.075030
3.	6.	0.	2.055448	-0.068244	0.371753
4.	6.	0.	0.520151	-0.201014	0.564365
5.	6.	0.	-0.287867	-0.818740	-0.608219
6.	6.	0.	0.341460	-2.164856	-1.033376
7.	6.	0.	-0.131606	0.165231	1.699853
8.	6.	0.	-1.611616	0.071631	1.882063
9.	6.	0.	-2.421880	-0.398275	0.756135
10.	6.	0.	-1.722313	-1.316464	-0.208389
11.	6.	0.	-3.677249	0.068777	0.614303
12.	6.	0.	-4.544928	-0.154975	-0.597056
13.	6.	0.	-3.629392	-0.547892	-1.785772
14.	6.	0.	-2.634879	-1.649653	-1.415033
15.	6.	0.	2.857759	0.294664	1.645560
16.	6.	0.	2.402780	0.994341	-0.712324
17.	8.	0.	2.226947	2.345473	-0.198083
18.	6.	0.	1.151646	3.077213	-0.515989
19.	6.	0.	1.266645	4.497808	0.007640
20.	6.	0.	-0.105847	5.070302	0.382296
21.	6.	0.	1.978577	5.364092	-1.056175
22.	8.	0.	0.207894	2.663337	-1.181266
23.	8.	0.	3.986706	-1.344451	-0.426969
24.	6.	0.	4.833710	-2.338970	-0.079273
25.	6.	0.	6.239566	-2.037653	-0.525852

26.	8.	0.	4.485317	-3.341514	0.520880
27.	6.	0.	-0.185267	-3.120301	0.044066
28.	8.	0.	-1.428401	-2.546035	0.516734
29.	6.	0.	-5.287231	1.163982	-0.930318
30.	6.	0.	-5.593852	-1.233744	-0.355813
31.	6.	0.	-5.722913	-2.024654	0.713914
32.	8.	0.	-2.072691	0.472507	2.960113
33.	8.	0.	0.455872	0.698883	2.801161
34.	8.	0.	-0.328345	0.035159	-1.743194
35.	1.	0.	2.250114	-3.105822	-1.425762
36.	1.	0.	2.049809	-1.518445	-2.157882
37.	1.	0.	2.521284	-2.127927	0.796498
38.	1.	0.	-0.110410	-2.433888	-1.991828
39.	1.	0.	-4.072804	0.707230	1.402481
40.	1.	0.	-4.246353	-0.879614	-2.629328
41.	1.	0.	-3.088173	0.345837	-2.116373
42.	1.	0.	-3.185770	-2.553026	-1.132616
43.	1.	0.	-2.036507	-1.902082	-2.292490
44.	1.	0.	2.676788	-0.427541	2.443937
45.	1.	0.	2.612152	1.285061	2.019303
46.	1.	0.	3.922433	0.275568	1.396849
47.	1.	0.	3.467869	0.935861	-0.934802
48.	1.	0.	1.829990	0.875728	-1.628510
49.	1.	0.	1.904287	4.458408	0.896896
50.	1.	0.	-0.759510	5.127896	-0.493086
51.	1.	0.	0.016505	6.079781	0.786417
52.	1.	0.	-0.602592	4.457750	1.141114
53.	1.	0.	2.966995	4.965829	-1.303912
54.	1.	0.	1.384386	5.419807	-1.974388
55.	1.	0.	2.106869	6.380106	-0.670991
56.	1.	0.	6.894533	-2.868171	-0.264360
57.	1.	0.	6.261764	-1.871369	-1.606728
58.	1.	0.	6.593756	-1.121330	-0.043799
59.	1.	0.	0.490409	-3.210045	0.902067
60.	1.	0.	-0.379175	-4.123626	-0.349243
61.	1.	0.	-5.900129	1.035669	-1.828904
62.	1.	0.	-4.573512	1.972814	-1.118432
63.	1.	0.	-5.944308	1.465149	-0.107864
64.	1.	0.	-6.307797	-1.333909	-1.175068
65.	1.	0.	-6.523090	-2.757224	0.770867
66.	1.	0.	-5.043958	-1.974268	1.560930
67.	1.	0.	-0.286129	0.864809	3.422294
68.	1.	0.	-0.279439	0.975590	-1.474482

S2 Quantum chemical CD calculation of eutypellenoid B (**2**).

ECD calculation details

1. Methods

Monte Carlo conformational searches were carried out by means of the Spartan's 10 software using Merck Molecular Force Field (MMFF). The conformers with Boltzmann-population of over 5% were chosen for ECD calculations, and then the conformers were initially optimized at B3LYP/6-31+g (d, p) level in MeOH using the CPCM polarizable conductor calculation model. The theoretical calculation of ECD was conducted in MeOH using Time-dependent Density functional theory (TD-DFT) at the B3LYP/6-311+g (d, p) level for all conformers of compound **2**. Rotatory strengths for a total of 50 excited states were calculated. ECD spectra were generated using the program SpecDis 1.6 (University of Würzburg, Würzburg, Germany) and GraphPad Prism 5 (University of California San Diego, USA) from dipole-length rotational strengths by applying Gaussian band shapes with sigma = 0.3 eV.

2. Results

Table S1.2.1.Gibbs free energies[a] and equilibrium populations[b] of low-energy conformers of **2**.

Conformers	In MeOH	
	ΔG	P (%)
**2a**	0.05	39.93
**2b**	0	41.80
**2c**	0.67	11.84
**2d**	1.56	6.44

[a]B3LYP/6-31+G(d,p), in kcal/mol. [b]From ΔG values at 298.15K.

Table S1.2.2.Cartesian coordinates for the low-energy reoptimized MMFF conformers of **2** at B3LYP/6-311+G(d,p) level of theory in CH_3OH.

**2a**		Standard Orientation (Ångstroms)			
I	Atom	Type	X	Y	Z
1.	6.	0.	0.854966	2.297286	-1.570926
2.	6.	0.	1.884197	1.341235	-0.959283
3.	6.	0.	1.438597	-0.152228	-0.987051
4.	6.	0.	-0.061589	-0.275219	-0.676170
5.	6.	0.	-0.851063	0.807429	-0.528906
6.	6.	0.	-0.449260	2.231621	-0.771602
7.	6.	0.	-0.739753	-1.584045	-0.689724
8.	6.	0.	-2.226864	-1.621101	-0.698722
9.	6.	0.	-3.009455	-0.525576	-0.625432
10.	6.	0.	-2.339452	0.792480	-0.343625
11.	6.	0.	-4.517342	-0.493273	-0.566603
12.	6.	0.	-4.988511	-0.106944	0.888500
13.	6.	0.	-4.277564	1.214997	1.265257
14.	6.	0.	-2.749283	1.199273	1.105235

15.	8.	0.	2.095532	1.733884	0.431122
16.	6.	0.	3.098851	2.593070	0.710941
17.	6.	0.	3.154570	2.907551	2.182630
18.	8.	0.	3.845548	3.057679	-0.134234
19.	6.	0.	2.230785	-0.966065	0.057199
20.	6.	0.	1.727072	-0.706691	-2.407505
21.	6.	0.	-4.716804	-1.254934	1.844572
22.	6.	0.	-4.211010	-1.193807	3.080964
23.	8.	0.	3.645398	-0.773832	-0.204810
24.	6.	0.	4.509985	-1.432642	0.589891
25.	6.	0.	5.957856	-1.175492	0.204034
26.	8.	0.	4.151397	-2.157216	1.505184
27.	6.	0.	6.587142	-2.475926	-0.333034
28.	6.	0.	6.739321	-0.626752	1.410725
29.	8.	0.	-0.157763	-2.679354	-0.757431
30.	8.	0.	-2.730360	-2.890008	-0.782657
31.	6.	0.	-1.723094	2.796914	-1.447834
32.	8.	0.	-2.771958	1.802680	-1.286887
33.	6.	0.	-6.516995	0.128433	0.854944
34.	8.	0.	-5.158082	-1.662862	-1.067578
35.	1.	0.	0.675662	2.008688	-2.612103
36.	1.	0.	1.260801	3.312808	-1.582523
37.	1.	0.	2.840220	1.431183	-1.474331
38.	1.	0.	-0.298075	2.731423	0.193368
39.	1.	0.	-4.852256	0.326291	-1.214481
40.	1.	0.	-4.685816	2.011793	0.632762
41.	1.	0.	-4.529021	1.487223	2.296077
42.	1.	0.	-2.345381	2.193098	1.325078
43.	1.	0.	-2.286794	0.494612	1.803100
44.	1.	0.	4.080692	3.434345	2.411489
45.	1.	0.	2.303713	3.544446	2.446824
46.	1.	0.	3.080924	1.993098	2.776223
47.	1.	0.	2.013392	-0.630028	1.072102
48.	1.	0.	1.994522	-2.025880	-0.029672
49.	1.	0.	2.795928	-0.643668	-2.628037
50.	1.	0.	1.184194	-0.143011	-3.170866
51.	1.	0.	1.423095	-1.752838	-2.477822
52.	1.	0.	-5.033461	-2.228616	1.470579
53.	1.	0.	-3.884908	-0.266129	3.542956
54.	1.	0.	-4.110954	-2.092137	3.683924
55.	1.	0.	5.956437	-0.427041	-0.594171
56.	1.	0.	7.622180	-2.286211	-0.633561
57.	1.	0.	6.044628	-2.856071	-1.204655

58.	1.	0.	6.590315	-3.252718	0.438263
59.	1.	0.	6.740914	-1.349163	2.232824
60.	1.	0.	7.776762	-0.433412	1.120705
61.	1.	0.	6.309101	0.311101	1.776660
62.	1.	0.	-1.954465	-3.485661	-0.840721
63.	1.	0.	-1.582226	2.946537	-2.522484
64.	1.	0.	-2.047219	3.739944	-0.997291
65.	1.	0.	-6.866470	0.470004	1.834511
66.	1.	0.	-7.052081	-0.790535	0.600989
67.	1.	0.	-6.774439	0.891985	0.111403
68.	1.	0.	-4.579428	-2.430786	-0.939644

**2b**		Standard Orientation (Ångstroms)			
I	Atom	Type	X	Y	Z
1.	6.	0.	0.853892	2.306280	-1.558371
2.	6.	0.	1.885125	1.363367	-0.930205
3.	6.	0.	1.451294	-0.133707	-0.948468
4.	6.	0.	-0.050856	-0.265930	-0.651083
5.	6.	0.	-0.850286	0.811552	-0.520433
6.	6.	0.	-0.456834	2.236952	-0.770053
7.	6.	0.	-0.718477	-1.580239	-0.660575
8.	6.	0.	-2.205011	-1.629149	-0.685959
9.	6.	0.	-2.996923	-0.539338	-0.630012
10.	6.	0.	-2.340509	0.786284	-0.351263
11.	6.	0.	-4.505564	-0.518682	-0.588055
12.	6.	0.	-4.995733	-0.124609	0.858560
13.	6.	0.	-4.300185	1.206549	1.231658
14.	6.	0.	-2.770068	1.201762	1.089302
15.	8.	0.	2.082524	1.770314	0.458166
16.	6.	0.	3.082342	2.633269	0.738713
17.	6.	0.	3.126538	2.959971	2.208127
18.	8.	0.	3.835042	3.092123	-0.104328
19.	6.	0.	2.239930	-0.931586	0.110753
20.	6.	0.	1.757735	-0.698568	-2.361043
21.	6.	0.	-4.724181	-1.261974	1.827368
22.	6.	0.	-4.234941	-1.184903	3.069522
23.	8.	0.	3.654787	-0.728070	-0.137296
24.	6.	0.	4.518032	-1.378248	0.667201
25.	6.	0.	5.961591	-1.143382	0.251845
26.	8.	0.	4.156472	-2.090481	1.590567
27.	6.	0.	6.376848	-2.237006	-0.757488

28.	6.	0.	6.893403	-1.113487	1.469746
29.	8.	0.	-0.127209	-2.671437	-0.712174
30.	8.	0.	-2.697597	-2.902639	-0.765233
31.	6.	0.	-1.728574	2.788050	-1.461982
32.	8.	0.	-2.770091	1.785154	-1.307873
33.	6.	0.	-6.525787	0.096919	0.806707
34.	8.	0.	-5.131598	-1.697252	-1.086580
35.	1.	0.	0.685952	2.007962	-2.598706
36.	1.	0.	1.251949	3.324835	-1.574621
37.	1.	0.	2.844578	1.456039	-1.438296
38.	1.	0.	-0.317610	2.744861	0.192472
39.	1.	0.	-4.839727	0.293044	-1.246110
40.	1.	0.	-4.707729	1.994223	0.587391
41.	1.	0.	-4.565683	1.485889	2.257039
42.	1.	0.	-2.376517	2.200522	1.305520
43.	1.	0.	-2.310157	0.506520	1.798240
44.	1.	0.	4.051025	3.488528	2.439647
45.	1.	0.	2.273867	3.599275	2.460409
46.	1.	0.	3.048187	2.050534	2.808791
47.	1.	0.	2.007959	-0.589016	1.120268
48.	1.	0.	2.013845	-1.994194	0.030391
49.	1.	0.	2.828053	-0.628419	-2.571996
50.	1.	0.	1.217365	-0.146544	-3.134678
51.	1.	0.	1.463289	-1.747874	-2.424587
52.	1.	0.	-5.025748	-2.242071	1.457745
53.	1.	0.	-3.924592	-0.249779	3.527318
54.	1.	0.	-4.133025	-2.076849	3.681576
55.	1.	0.	5.996293	-0.175786	-0.259748
56.	1.	0.	7.400968	-2.052748	-1.096409
57.	1.	0.	5.723843	-2.244473	-1.635431
58.	1.	0.	6.344892	-3.227866	-0.291912
59.	1.	0.	6.875914	-2.070603	1.999538
60.	1.	0.	7.920033	-0.920074	1.143678
61.	1.	0.	6.606640	-0.327198	2.175236
62.	1.	0.	-1.916505	-3.492672	-0.809401
63.	1.	0.	-1.577944	2.934406	-2.535721
64.	1.	0.	-2.065685	3.730098	-1.018874
65.	1.	0.	-6.888911	0.443089	1.779668
66.	1.	0.	-7.049880	-0.828825	0.554481
67.	1.	0.	-6.781945	0.852173	0.054296
68.	1.	0.	-4.548435	-2.459574	-0.946286

2c		Standard Orientation (Ångstroms)			
I	Atom	Type	X	Y	Z
1.	6.	0.	1.046690	2.107356	-1.629335
2.	6.	0.	1.996317	1.162005	-0.886249
3.	6.	0.	1.501805	-0.314915	-0.858903
4.	6.	0.	-0.014322	-0.367131	-0.611498
5.	6.	0.	-0.768070	0.750151	-0.575715
6.	6.	0.	-0.312659	2.134928	-0.926096
7.	6.	0.	-0.740473	-1.649500	-0.566922
8.	6.	0.	-2.226637	-1.634851	-0.626319
9.	6.	0.	-2.969717	-0.509957	-0.643861
10.	6.	0.	-2.259044	0.799790	-0.424660
11.	6.	0.	-4.476555	-0.422234	-0.619592
12.	6.	0.	-4.960973	0.059404	0.803003
13.	6.	0.	-4.208239	1.373072	1.122968
14.	6.	0.	-2.678957	1.293918	0.995152
15.	8.	0.	2.117928	1.626403	0.493186
16.	6.	0.	3.071393	2.537881	0.780581
17.	6.	0.	3.039559	2.923013	2.236112
18.	8.	0.	3.843258	2.990164	-0.048564
19.	6.	0.	2.213798	-1.097714	0.264528
20.	6.	0.	1.834993	-0.958070	-2.230761
21.	6.	0.	-4.751576	-1.043387	1.825507
22.	6.	0.	-4.262646	-0.933530	3.065313
23.	8.	0.	3.645248	-0.979168	0.060843
24.	6.	0.	4.440804	-1.692899	0.878760
25.	6.	0.	5.918254	-1.493667	0.577872
26.	8.	0.	4.004776	-2.410379	1.766809
27.	6.	0.	6.354930	-0.054884	0.916539
28.	6.	0.	6.259951	-1.863777	-0.876692
29.	8.	0.	-0.197907	-2.766801	-0.545236
30.	8.	0.	-2.774278	-2.888240	-0.647865
31.	6.	0.	-1.520371	2.632708	-1.753131
32.	8.	0.	-2.650068	1.782063	-1.415697
33.	6.	0.	-6.478453	0.349003	0.724716
34.	8.	0.	-5.148241	-1.594335	-1.071684
35.	1.	0.	0.932740	1.755757	-2.660215
36.	1.	0.	1.487725	3.106493	-1.677604
37.	1.	0.	2.990298	1.192152	-1.332431
38.	1.	0.	-0.233010	2.732286	-0.008897
39.	1.	0.	-4.769389	0.372104	-1.317176
40.	1.	0.	-4.575012	2.148836	0.440999

41.	1.	0.	-4.468751	1.709358	2.132476
42.	1.	0.	-2.242133	2.282652	1.169775
43.	1.	0.	-2.256306	0.609241	1.736816
44.	1.	0.	3.918867	3.519596	2.477826
45.	1.	0.	2.137735	3.511589	2.434782
46.	1.	0.	2.999961	2.032931	2.869041
47.	1.	0.	1.966217	-0.694940	1.248009
48.	1.	0.	1.935408	-2.149653	0.226003
49.	1.	0.	2.912984	-0.930658	-2.409763
50.	1.	0.	1.335882	-0.431171	-3.048056
51.	1.	0.	1.509966	-2.000173	-2.250878
52.	1.	0.	-5.102817	-2.022951	1.500932
53.	1.	0.	-3.905189	0.004710	3.480299
54.	1.	0.	-4.209261	-1.799813	3.718980
55.	1.	0.	6.436068	-2.184409	1.250654
56.	1.	0.	7.435833	0.042079	0.773505
57.	1.	0.	6.127953	0.199414	1.957030
58.	1.	0.	5.856196	0.670519	0.267095
59.	1.	0.	5.772897	-1.183672	-1.581591
60.	1.	0.	7.342105	-1.794356	-1.026226
61.	1.	0.	5.950800	-2.886811	-1.114553
62.	1.	0.	-2.019423	-3.512964	-0.641175
63.	1.	0.	-1.335131	2.532906	-2.828075
64.	1.	0.	-1.784175	3.670690	-1.531927
65.	1.	0.	-6.831654	0.761728	1.675143
66.	1.	0.	-7.044579	-0.562591	0.515286
67.	1.	0.	-6.692851	1.075682	-0.067785
68.	1.	0.	-4.604495	-2.374508	-0.880541

**2d**		Standard Orientation (Ångstroms)			
I	Atom	Type	X	Y	Z
1.	6.	0.	1.383121	-3.112335	0.146347
2.	6.	0.	2.383570	-2.015301	0.528700
3.	6.	0.	1.779956	-0.915820	1.453056
4.	6.	0.	0.335048	-0.596314	1.054018
5.	6.	0.	-0.310523	-1.284181	0.092853
6.	6.	0.	0.205051	-2.500521	-0.617067
7.	6.	0.	-0.462215	0.381256	1.811927
8.	6.	0.	-1.933523	0.427064	1.595174
9.	6.	0.	-2.587452	-0.336899	0.696762
10.	6.	0.	-1.757871	-1.136740	-0.272076

11.	6.	0.	-4.066109	-0.310933	0.393018
12.	6.	0.	-4.316346	0.366647	-1.009376
13.	6.	0.	-3.434035	-0.375227	-2.041083
14.	6.	0.	-1.946034	-0.472876	-1.670693
15.	8.	0.	2.869864	-1.391809	-0.696398
16.	6.	0.	3.978120	-1.899390	-1.277117
17.	6.	0.	4.314775	-1.164768	-2.547694
18.	8.	0.	4.607957	-2.840653	-0.823184
19.	6.	0.	2.670962	0.346549	1.467436
20.	6.	0.	1.814336	-1.479964	2.901850
21.	6.	0.	-4.050159	1.859017	-0.922183
22.	6.	0.	-3.398152	2.629825	-1.799100
23.	8.	0.	2.357861	1.199283	0.340534
24.	6.	0.	3.088592	2.320564	0.204294
25.	6.	0.	2.603898	3.176329	-0.954906
26.	8.	0.	4.008421	2.611045	0.954330
27.	6.	0.	3.782511	3.817927	-1.699397
28.	6.	0.	1.624098	4.241385	-0.415221
29.	8.	0.	-0.002673	1.141570	2.679622
30.	8.	0.	-2.567057	1.327909	2.406323
31.	6.	0.	-1.082920	-3.356459	-0.731622
32.	8.	0.	-2.194133	-2.517485	-0.312029
33.	6.	0.	-5.805776	0.173716	-1.380356
34.	8.	0.	-4.877504	0.236050	1.428378
35.	1.	0.	1.026635	-3.602059	1.058826
36.	1.	0.	1.889455	-3.875475	-0.451473
37.	1.	0.	3.246072	-2.454551	1.033685
38.	1.	0.	0.543904	-2.216723	-1.621503
39.	1.	0.	-4.393447	-1.352247	0.281670
40.	1.	0.	-3.831158	-1.390021	-2.159401
41.	1.	0.	-3.531767	0.104574	-3.020900
42.	1.	0.	-1.417290	-1.066155	-2.424191
43.	1.	0.	-1.479689	0.516700	-1.646684
44.	1.	0.	5.302900	-1.465137	-2.895525
45.	1.	0.	3.572095	-1.410584	-3.314028
46.	1.	0.	4.280554	-0.084285	-2.388108
47.	1.	0.	2.494162	0.917002	2.379472
48.	1.	0.	3.727154	0.067399	1.424182
49.	1.	0.	2.850091	-1.620025	3.229310
50.	1.	0.	1.305767	-2.445079	2.963842
51.	1.	0.	1.327802	-0.791765	3.594866
52.	1.	0.	-4.504236	2.342440	-0.057168
53.	1.	0.	-2.928744	2.238699	-2.697562

54.	1.	0.	-3.318355	3.702056	-1.642517
55.	1.	0.	2.055329	2.518253	-1.636529
56.	1.	0.	3.408859	4.412437	-2.538903
57.	1.	0.	4.466251	3.060690	-2.096206
58.	1.	0.	4.350592	4.477981	-1.036959
59.	1.	0.	2.130741	4.916226	0.282809
60.	1.	0.	1.236459	4.838214	-1.246580
61.	1.	0.	0.774398	3.783226	0.100132
62.	1.	0.	-1.869500	1.719297	2.972370
63.	1.	0.	-1.062697	-4.220417	-0.061165
64.	1.	0.	-1.257168	-3.705192	-1.754325
65.	1.	0.	-5.998079	0.570472	-2.382374
66.	1.	0.	-6.458626	0.693725	-0.674158
67.	1.	0.	-6.071936	-0.889982	-1.373764
68.	1.	0.	-4.365946	0.887712	1.932994

S3 Quantum chemical CD calculation of eutypellenone C (**3**).

ECD calculation details

1. Methods

Monte Carlo conformational searches were carried out by means of the Spartan's 10 software using Merck Molecular Force Field (MMFF). The conformers with Boltzmann-population of over 5% were chosen for ECD calculations, and then the conformers were initially optimized at B3LYP/6-31+g (d, p) level in MeOH using the CPCM polarizable conductor calculation model. The theoretical calculation of ECD was conducted in MeOH using Time-dependent Density functional theory (TD-DFT) at the B3LYP/6-311+g (d, p) level for all conformers of compounds **3**. Rotatory strengths for a total of 50 excited states were calculated. ECD spectra were generated using the program SpecDis 1.6 (University of Würzburg, Würzburg, Germany) and GraphPad Prism 5 (University of California San Diego, USA) from dipole-length rotational strengths by applying Gaussian band shapes with sigma = 0.3 eV.

2. Results

Table S1.2.1.Gibbs free energies[a] and equilibrium populations[b] of low-energy conformers of **3**.

Conformers	In MeOH	
	Δ*G*	*P* (%)
**3a**		18.64
**3b**		20.13
**3c**		19.45
**3d**		16.20
**3e**		19.74
**3f**		5.84

[a]B3LYP/6-31+G(d,p), in kcal/mol. [b]From Δ*G* values at 298.15K.

Table S1.2.2.Cartesian coordinates for the low-energy reoptimized MMFF conformers of **3** at B3LYP/6-311+G(d,p) level of theory in CH_3OH.

**3a**		Standard Orientation (Ångstroms)			
I	Atom	Type	X	Y	Z
1.	6.	0.	0.642538	2.302600	-1.506197
2.	6.	0.	1.695919	1.354426	-0.924429
3.	6.	0.	1.291746	-0.148671	-1.005829
4.	6.	0.	-0.208397	-0.325248	-0.716133
5.	6.	0.	-1.027758	0.729405	-0.531951
6.	6.	0.	-0.660895	2.173127	-0.713782
7.	6.	0.	-0.848730	-1.653381	-0.801170
8.	6.	0.	-2.331889	-1.732912	-0.843777
9.	6.	0.	-3.142738	-0.662111	-0.714360
10.	6.	0.	-2.518771	0.664467	-0.383480
11.	6.	0.	-4.639850	-0.719449	-0.659654
12.	6.	0.	-5.177720	-0.315870	0.755480
13.	6.	0.	-4.518000	1.019802	1.168507

14.	6.	0.	-2.984423	1.037299	1.056885
15.	6.	0.	1.611032	-0.645473	-2.441242
16.	6.	0.	2.095760	-0.976734	0.017559
17.	8.	0.	3.507869	-0.740330	-0.223452
18.	6.	0.	4.382215	-1.425990	0.536607
19.	6.	0.	5.825954	-1.090514	0.197712
20.	6.	0.	6.601620	-2.373290	-0.148560
21.	6.	0.	6.472941	-0.339555	1.378104
22.	8.	0.	4.035169	-2.216204	1.400840
23.	8.	0.	1.894811	1.704739	0.479504
24.	6.	0.	2.873723	2.580532	0.790545
25.	6.	0.	2.955247	2.802628	2.277865
26.	8.	0.	3.595108	3.109732	-0.038672
27.	6.	0.	-1.948155	2.736054	-1.370120
28.	8.	0.	-2.948483	1.683508	-1.323153
29.	6.	0.	-6.710055	-0.129888	0.656516
30.	6.	0.	-4.920738	-1.457331	1.728030
31.	6.	0.	-4.454994	-1.390894	2.979622
32.	8.	0.	-0.225489	-2.724349	-0.904410
33.	8.	0.	-2.807495	-2.999329	-1.016854
34.	1.	0.	0.473346	2.044837	-2.557268
35.	1.	0.	1.020646	3.328633	-1.481754
36.	1.	0.	2.650259	1.488462	-1.433093
37.	1.	0.	-0.521909	2.637420	0.270456
38.	1.	0.	-5.050035	-0.009722	-1.389360
39.	1.	0.	-4.999667	-1.716360	-0.927608
40.	1.	0.	-4.922442	1.810379	0.525901
41.	1.	0.	-4.807118	1.280723	2.192868
42.	1.	0.	-2.608995	2.034511	1.310303
43.	1.	0.	-2.530141	0.330219	1.758035
44.	1.	0.	1.061569	-0.069863	-3.190866
45.	1.	0.	1.335780	-1.695953	-2.550999
46.	1.	0.	2.680218	-0.546290	-2.646817
47.	1.	0.	1.861114	-0.681586	1.041395
48.	1.	0.	1.887101	-2.038253	-0.108449
49.	1.	0.	5.812268	-0.431327	-0.675592
50.	1.	0.	6.622035	-3.057125	0.705604
51.	1.	0.	7.633453	-2.120260	-0.411219
52.	1.	0.	6.153396	-2.897356	-0.998940
53.	1.	0.	7.504951	-0.077239	1.125274
54.	1.	0.	5.935185	0.584954	1.611398
55.	1.	0.	6.488087	-0.966627	2.275311
56.	1.	0.	3.692092	3.575447	2.494885

57.	1.	0.	1.977954	3.095896	2.671154
58.	1.	0.	3.244412	1.870348	2.773053
59.	1.	0.	-1.796134	2.988241	-2.423691
60.	1.	0.	-2.323648	3.620535	-0.845397
61.	1.	0.	-7.201901	-1.063954	0.362826
62.	1.	0.	-7.123784	0.181036	1.621510
63.	1.	0.	-6.958713	0.634285	-0.088728
64.	1.	0.	-5.204671	-2.437451	1.340418
65.	1.	0.	-4.153634	-0.458983	3.449683
66.	1.	0.	-4.361887	-2.287947	3.585555
67.	1.	0.	-2.014597	-3.569944	-1.072763

**3b**		Standard Orientation (Ångstroms)			
I	Atom	Type	X	Y	Z
1.	6.	0.	0.637365	2.305605	-1.512877
2.	6.	0.	1.692720	1.373815	-0.908884
3.	6.	0.	1.303418	-0.133709	-0.977618
4.	6.	0.	-0.197616	-0.320777	-0.699261
5.	6.	0.	-1.028226	0.728472	-0.535639
6.	6.	0.	-0.673391	2.173130	-0.733127
7.	6.	0.	-0.824981	-1.655675	-0.773644
8.	6.	0.	-2.306979	-1.749530	-0.826709
9.	6.	0.	-3.128807	-0.684971	-0.716301
10.	6.	0.	-2.519746	0.651754	-0.398357
11.	6.	0.	-4.625696	-0.756562	-0.670895
12.	6.	0.	-5.177042	-0.339703	0.735054
13.	6.	0.	-4.534324	1.008607	1.133487
14.	6.	0.	-3.000224	1.039813	1.033123
15.	6.	0.	1.640047	-0.643637	-2.404402
16.	6.	0.	2.105973	-0.941778	0.062861
17.	8.	0.	3.517701	-0.694321	-0.167980
18.	6.	0.	4.391215	-1.339595	0.628081
19.	6.	0.	5.833625	-1.052543	0.242843
20.	6.	0.	6.384031	-2.244069	-0.569642
21.	6.	0.	6.687993	-0.775104	1.488554
22.	8.	0.	4.042803	-2.085227	1.530311
23.	8.	0.	1.871757	1.741858	0.493240
24.	6.	0.	2.841444	2.626899	0.806685
25.	6.	0.	2.873482	2.900967	2.287264
26.	8.	0.	3.580355	3.139882	-0.017318
27.	6.	0.	-1.959000	2.714007	-1.410800

28.	8.	0.	-2.951231	1.654187	-1.355042
29.	6.	0.	-6.710668	-0.171921	0.624187
30.	6.	0.	-4.913577	-1.464497	1.725222
31.	6.	0.	-4.458985	-1.374348	2.979430
32.	8.	0.	-0.191062	-2.721888	-0.859357
33.	8.	0.	-2.769425	-3.022432	-0.987435
34.	1.	0.	0.482247	2.034605	-2.562739
35.	1.	0.	1.005802	3.335360	-1.495976
36.	1.	0.	2.651839	1.511105	-1.407564
37.	1.	0.	-0.549881	2.651771	0.246249
38.	1.	0.	-5.038146	-0.060888	-1.412755
39.	1.	0.	-4.973608	-1.760592	-0.927760
40.	1.	0.	-4.941896	1.785568	0.476392
41.	1.	0.	-4.833826	1.281436	2.151744
42.	1.	0.	-2.636568	2.044125	1.275479
43.	1.	0.	-2.544405	0.347043	1.747455
44.	1.	0.	1.089280	-0.084268	-3.165321
45.	1.	0.	1.378604	-1.698735	-2.503286
46.	1.	0.	2.709459	-0.533723	-2.603018
47.	1.	0.	1.858372	-0.636588	1.080565
48.	1.	0.	1.909106	-2.006984	-0.052123
49.	1.	0.	5.828823	-0.166952	-0.400318
50.	1.	0.	6.391707	-3.156231	0.036070
51.	1.	0.	7.411340	-2.032002	-0.881698
52.	1.	0.	5.787416	-2.429518	-1.468428
53.	1.	0.	7.717860	-0.558568	1.188208
54.	1.	0.	6.310211	0.084638	2.051186
55.	1.	0.	6.699556	-1.642964	2.154887
56.	1.	0.	3.765973	3.474703	2.535961
57.	1.	0.	1.984711	3.475710	2.568048
58.	1.	0.	2.854642	1.965478	2.852022
59.	1.	0.	-1.798759	2.949264	-2.467090
60.	1.	0.	-2.346769	3.604253	-0.905098
61.	1.	0.	-7.190556	-1.115346	0.340775
62.	1.	0.	-7.133617	0.147961	1.582237
63.	1.	0.	-6.963040	0.578943	-0.133250
64.	1.	0.	-5.181724	-2.453511	1.349205
65.	1.	0.	-4.173479	-0.431904	3.438224
66.	1.	0.	-4.359330	-2.261330	3.598991
67.	1.	0.	-1.970849	-3.586215	-1.030431

**3c**		Standard Orientation (Ångstroms)			
I	Atom	Type	X	Y	Z
1.	6.	0.	0.639114	2.322826	-1.482733
2.	6.	0.	1.696498	1.388130	-0.887472
3.	6.	0.	1.300950	-0.117377	-0.956412
4.	6.	0.	-0.197571	-0.298997	-0.661545
5.	6.	0.	-1.022696	0.752072	-0.482610
6.	6.	0.	-0.666187	2.195384	-0.693019
7.	6.	0.	-0.830854	-1.630119	-0.740123
8.	6.	0.	-2.313250	-1.718581	-0.772563
9.	6.	0.	-3.133826	-0.658705	-0.616511
10.	6.	0.	-2.514677	0.682221	-0.332932
11.	6.	0.	-4.632396	-0.738675	-0.536373
12.	6.	0.	-5.194301	-0.213373	0.820570
13.	6.	0.	-4.531646	1.153696	1.138317
14.	6.	0.	-2.998127	1.121873	1.081282
15.	6.	0.	1.621431	-0.624334	-2.388007
16.	6.	0.	2.110127	-0.932006	0.073731
17.	8.	0.	3.520766	-0.693763	-0.172842
18.	6.	0.	4.398680	-1.348748	0.610401
19.	6.	0.	5.838596	-1.071526	0.209135
20.	6.	0.	6.371093	-2.265732	-0.611273
21.	6.	0.	6.709009	-0.802279	1.445578
22.	8.	0.	4.055161	-2.095023	1.513975
23.	8.	0.	1.886637	1.752787	0.514043
24.	6.	0.	2.863761	2.631073	0.823344
25.	6.	0.	2.907884	2.899984	2.304596
26.	8.	0.	3.600016	3.142195	-0.004178
27.	6.	0.	-1.956016	2.730248	-1.369023
28.	8.	0.	-2.937937	1.660446	-1.320821
29.	6.	0.	-6.721580	-0.013781	0.681861
30.	6.	0.	-4.963223	-1.160442	1.986724
31.	6.	0.	-4.492913	-2.412158	1.973174
32.	8.	0.	-0.202314	-2.697954	-0.844059
33.	8.	0.	-2.778956	-2.989542	-0.940154
34.	1.	0.	0.475309	2.051975	-2.531280
35.	1.	0.	1.010243	3.351680	-1.469201
36.	1.	0.	2.652408	1.523262	-1.392841
37.	1.	0.	-0.536695	2.682375	0.281273
38.	1.	0.	-5.056018	-0.104924	-1.326675
39.	1.	0.	-4.975304	-1.758836	-0.724227
40.	1.	0.	-4.896689	1.897422	0.420645

41.	1.	0.	-4.848458	1.491116	2.132546
42.	1.	0.	-2.595918	2.113631	1.312691
43.	1.	0.	-2.593707	0.424221	1.823592
44.	1.	0.	1.062823	-0.062916	-3.141634
45.	1.	0.	1.358377	-1.678986	-2.486586
46.	1.	0.	2.688663	-0.514487	-2.598004
47.	1.	0.	1.876258	-0.626480	1.094560
48.	1.	0.	1.904796	-1.995716	-0.040423
49.	1.	0.	5.832912	-0.184824	-0.432489
50.	1.	0.	6.379089	-3.178797	-0.006946
51.	1.	0.	7.396250	-2.060586	-0.934793
52.	1.	0.	5.762833	-2.445463	-1.503388
53.	1.	0.	7.736990	-0.592762	1.133979
54.	1.	0.	6.343954	0.059290	2.013817
55.	1.	0.	6.721752	-1.671287	2.110414
56.	1.	0.	3.804617	3.469050	2.548701
57.	1.	0.	2.023634	3.477334	2.594118
58.	1.	0.	2.889395	1.962537	2.866144
59.	1.	0.	-1.798218	2.972683	-2.423801
60.	1.	0.	-2.351532	3.614264	-0.858090
61.	1.	0.	-7.213859	-0.954777	0.413994
62.	1.	0.	-7.157141	0.339315	1.623182
63.	1.	0.	-6.945778	0.726515	-0.094557
64.	1.	0.	-5.262749	-0.736793	2.947928
65.	1.	0.	-4.166287	-2.912508	1.066694
66.	1.	0.	-4.414809	-2.979306	2.897165
67.	1.	0.	-1.981413	-3.554437	-0.992945

**3d**		Standard Orientation (Ångstroms)			
I	Atom	Type	X	Y	Z
1.	6.	0.	0.712348	2.351800	-1.439415
2.	6.	0.	1.751906	1.382011	-0.868291
3.	6.	0.	1.334848	-0.115122	-0.984376
4.	6.	0.	-0.168947	-0.283436	-0.709235
5.	6.	0.	-0.979965	0.774787	-0.509255
6.	6.	0.	-0.599130	2.218350	-0.660962
7.	6.	0.	-0.820761	-1.603440	-0.823864
8.	6.	0.	-2.304825	-1.668361	-0.874504
9.	6.	0.	-3.107559	-0.593174	-0.728793
10.	6.	0.	-2.472398	0.721085	-0.369900
11.	6.	0.	-4.605971	-0.642454	-0.683202

12.	6.	0.	-5.160310	-0.255653	0.734846
13.	6.	0.	-4.477475	1.073684	1.171723
14.	6.	0.	-2.943998	1.074022	1.074631
15.	6.	0.	1.660399	-0.585223	-2.427271
16.	6.	0.	2.122624	-0.971766	0.028116
17.	8.	0.	3.539049	-0.744365	-0.195796
18.	6.	0.	4.398819	-1.446924	0.565435
19.	6.	0.	5.849624	-1.145322	0.225419
20.	6.	0.	6.521782	-2.410193	-0.342275
21.	6.	0.	6.587196	-0.624842	1.472216
22.	8.	0.	4.034994	-2.237632	1.422242
23.	8.	0.	1.941036	1.700988	0.544330
24.	6.	0.	2.927198	2.557991	0.883725
25.	6.	0.	2.970816	2.778262	2.372963
26.	8.	0.	3.671242	3.086591	0.074417
27.	6.	0.	-1.875955	2.804335	-1.317327
28.	8.	0.	-2.888105	1.761962	-1.291193
29.	6.	0.	-6.676069	-0.030281	0.627474
30.	6.	0.	-4.812919	-1.360665	1.717837
31.	6.	0.	-5.651237	-2.026504	2.520437
32.	8.	0.	-0.207534	-2.678184	-0.944102
33.	8.	0.	-2.791109	-2.927092	-1.068186
34.	1.	0.	0.550118	2.117633	-2.497120
35.	1.	0.	1.099537	3.373582	-1.390317
36.	1.	0.	2.712014	1.517674	-1.365523
37.	1.	0.	-0.464881	2.662928	0.332959
38.	1.	0.	-5.004920	0.084036	-1.402836
39.	1.	0.	-4.970624	-1.631657	-0.970418
40.	1.	0.	-4.865167	1.880322	0.538225
41.	1.	0.	-4.771207	1.308771	2.201244
42.	1.	0.	-2.561654	2.063624	1.346408
43.	1.	0.	-2.498623	0.357035	1.772894
44.	1.	0.	1.122541	0.011497	-3.168680
45.	1.	0.	1.375576	-1.630349	-2.560920
46.	1.	0.	2.732070	-0.492611	-2.622598
47.	1.	0.	1.882030	-0.695797	1.055884
48.	1.	0.	1.905036	-2.028445	-0.121609
49.	1.	0.	5.851238	-0.366658	-0.543327
50.	1.	0.	6.523233	-3.214660	0.400022
51.	1.	0.	7.559384	-2.187073	-0.609216
52.	1.	0.	6.009721	-2.769662	-1.240725
53.	1.	0.	7.626756	-0.396929	1.216959
54.	1.	0.	6.125400	0.288350	1.861322

55.	1.	0.	6.585277	-1.377716	2.266589
56.	1.	0.	3.864417	3.344117	2.635180
57.	1.	0.	2.082979	3.339054	2.683047
58.	1.	0.	2.959487	1.822062	2.902385
59.	1.	0.	-1.714393	3.069218	-2.366342
60.	1.	0.	-2.245638	3.685311	-0.782796
61.	1.	0.	-7.192625	-0.937846	0.297211
62.	1.	0.	-7.102319	0.272109	1.589877
63.	1.	0.	-6.889657	0.762842	-0.096336
64.	1.	0.	-3.757030	-1.626552	1.762950
65.	1.	0.	-6.720356	-1.837230	2.545085
66.	1.	0.	-5.281106	-2.798427	3.189612
67.	1.	0.	-2.003446	-3.504490	-1.129709

**3e**		Standard Orientation (Ångstroms)			
I	Atom	Type	X	Y	Z
1.	6.	0.	0.640141	2.330672	-1.469678
2.	6.	0.	1.697624	1.399865	-0.868608
3.	6.	0.	1.307428	-0.106942	-0.938076
4.	6.	0.	-0.192103	-0.293328	-0.651446
5.	6.	0.	-1.021611	0.755082	-0.477148
6.	6.	0.	-0.668762	2.199433	-0.686608
7.	6.	0.	-0.820507	-1.626597	-0.732480
8.	6.	0.	-2.302399	-1.720009	-0.772774
9.	6.	0.	-3.127294	-0.662826	-0.621363
10.	6.	0.	-2.514128	0.680318	-0.335216
11.	6.	0.	-4.625961	-0.747899	-0.548739
12.	6.	0.	-5.196461	-0.224045	0.805143
13.	6.	0.	-4.540422	1.145641	1.125322
14.	6.	0.	-3.006540	1.119226	1.076139
15.	6.	0.	1.638162	-0.614410	-2.367108
16.	6.	0.	2.113156	-0.918047	0.097591
17.	8.	0.	3.523988	-0.670905	-0.136239
18.	6.	0.	4.399765	-1.336614	0.641085
19.	6.	0.	5.838739	-1.054443	0.239219
20.	6.	0.	6.278277	-2.096479	-0.813492
21.	6.	0.	6.767889	-1.056208	1.459527
22.	8.	0.	4.052724	-2.095752	1.532266
23.	8.	0.	1.880210	1.766693	0.533344
24.	6.	0.	2.856801	2.644154	0.846463
25.	6.	0.	2.893153	2.916375	2.327302

26.	8.	0.	3.597790	3.153557	0.022074
27.	6.	0.	-1.956842	2.729560	-1.369706
28.	8.	0.	-2.935440	1.656528	-1.326006
29.	6.	0.	-6.723790	-0.030234	0.658978
30.	6.	0.	-4.967462	-1.169579	1.972947
31.	6.	0.	-4.492614	-2.419606	1.962326
32.	8.	0.	-0.187908	-2.692428	-0.832267
33.	8.	0.	-2.762955	-2.992602	-0.942157
34.	1.	0.	0.482573	2.058687	-2.518894
35.	1.	0.	1.007813	3.360763	-1.454855
36.	1.	0.	2.655431	1.537631	-1.369604
37.	1.	0.	-0.545834	2.687549	0.287965
38.	1.	0.	-5.047764	-0.115873	-1.341389
39.	1.	0.	-4.964394	-1.769313	-0.737920
40.	1.	0.	-4.904483	1.887595	0.405320
41.	1.	0.	-4.863443	1.482587	2.117717
42.	1.	0.	-2.609082	2.112563	1.308982
43.	1.	0.	-2.603504	0.423552	1.821056
44.	1.	0.	1.083391	-0.054818	-3.124931
45.	1.	0.	1.378033	-1.669707	-2.466471
46.	1.	0.	2.706561	-0.502485	-2.570014
47.	1.	0.	1.868664	-0.615128	1.116824
48.	1.	0.	1.914571	-1.982698	-0.019415
49.	1.	0.	5.854174	-0.065951	-0.231730
50.	1.	0.	6.267454	-3.105960	-0.388761
51.	1.	0.	7.298413	-1.876450	-1.142969
52.	1.	0.	5.626248	-2.082121	-1.692086
53.	1.	0.	7.790996	-0.829127	1.144268
54.	1.	0.	6.464365	-0.305618	2.196195
55.	1.	0.	6.768606	-2.034591	1.949262
56.	1.	0.	3.800244	3.465886	2.578081
57.	1.	0.	2.020253	3.516411	2.605307
58.	1.	0.	2.846685	1.982209	2.892419
59.	1.	0.	-1.794269	2.971846	-2.423793
60.	1.	0.	-2.358013	3.612582	-0.861455
61.	1.	0.	-7.211325	-0.973267	0.389603
62.	1.	0.	-7.165107	0.322049	1.597926
63.	1.	0.	-6.947032	0.708567	-0.119138
64.	1.	0.	-5.273003	-0.746443	2.932480
65.	1.	0.	-4.159894	-2.919215	1.057656
66.	1.	0.	-4.416781	-2.985958	2.886996
67.	1.	0.	-1.963240	-3.554847	-0.990346

3f		Standard Orientation (Ångstroms)			
I	Atom	Type	X	Y	Z
1.	6.	0.	0.821962	2.115212	-1.564898
2.	6.	0.	1.807216	1.185577	-0.848897
3.	6.	0.	1.361981	-0.307490	-0.851479
4.	6.	0.	-0.154751	-0.419995	-0.621568
5.	6.	0.	-0.948304	0.668407	-0.563408
6.	6.	0.	-0.530847	2.081025	-0.849163
7.	6.	0.	-0.832473	-1.732160	-0.633550
8.	6.	0.	-2.314688	-1.773342	-0.730002
9.	6.	0.	-3.096846	-0.673894	-0.720733
10.	6.	0.	-2.445729	0.658440	-0.475612
11.	6.	0.	-4.596167	-0.687802	-0.719158
12.	6.	0.	-5.170752	-0.157845	0.638321
13.	6.	0.	-4.492387	1.193223	0.960979
14.	6.	0.	-2.955659	1.164920	0.908415
15.	6.	0.	1.728223	-0.908897	-2.234462
16.	6.	0.	2.092561	-1.088093	0.260685
17.	8.	0.	3.521577	-0.926477	0.064583
18.	6.	0.	4.333794	-1.633450	0.871224
19.	6.	0.	5.806247	-1.397558	0.571708
20.	6.	0.	6.209842	0.051367	0.907238
21.	6.	0.	6.156330	-1.762636	-0.882333
22.	8.	0.	3.915576	-2.375177	1.748054
23.	8.	0.	1.924266	1.626033	0.539172
24.	6.	0.	2.856435	2.554347	0.840354
25.	6.	0.	2.822784	2.909304	2.303604
26.	8.	0.	3.613000	3.042468	0.017263
27.	6.	0.	-1.759355	2.606613	-1.634854
28.	8.	0.	-2.807464	1.607620	-1.512709
29.	6.	0.	-6.693999	0.051980	0.471585
30.	6.	0.	-4.971498	-1.219092	1.709937
31.	6.	0.	-4.548543	-1.056162	2.967848
32.	8.	0.	-0.239677	-2.825164	-0.631587
33.	8.	0.	-2.822108	-3.036143	-0.819446
34.	1.	0.	0.710118	1.782897	-2.602505
35.	1.	0.	1.229426	3.129558	-1.593531
36.	1.	0.	2.796901	1.257680	-1.299855
37.	1.	0.	-0.444672	2.629409	0.097159
38.	1.	0.	-4.959691	-0.028371	-1.517484
39.	1.	0.	-4.975027	-1.693285	-0.920704
40.	1.	0.	-4.851498	1.934091	0.237247

41.	1.	0.	-4.812237	1.549083	1.947017
42.	1.	0.	-2.562546	2.171155	1.088959
43.	1.	0.	-2.547779	0.510912	1.685635
44.	1.	0.	1.216691	-0.381197	-3.043763
45.	1.	0.	1.440012	-1.960802	-2.280123
46.	1.	0.	2.805852	-0.839385	-2.404452
47.	1.	0.	1.830822	-0.709196	1.249937
48.	1.	0.	1.844311	-2.146620	0.202833
49.	1.	0.	6.340206	-2.075097	1.245315
50.	1.	0.	5.695010	0.764171	0.256457
51.	1.	0.	7.288288	0.172459	0.763976
52.	1.	0.	5.977183	0.302597	1.947211
53.	1.	0.	7.236439	-1.667379	-1.032486
54.	1.	0.	5.872047	-2.793408	-1.117867
55.	1.	0.	5.652732	-1.096099	-1.588619
56.	1.	0.	3.689157	3.521236	2.553584
57.	1.	0.	1.908565	3.472689	2.518032
58.	1.	0.	2.806855	2.006026	2.918621
59.	1.	0.	-1.543504	2.718843	-2.701607
60.	1.	0.	-2.121087	3.563214	-1.244296
61.	1.	0.	-7.198526	-0.892501	0.239215
62.	1.	0.	-7.131315	0.451414	1.392585
63.	1.	0.	-6.899297	0.756187	-0.342777
64.	1.	0.	-5.261491	-2.223095	1.394946
65.	1.	0.	-4.245132	-0.093186	3.368760
66.	1.	0.	-4.495182	-1.900460	3.649644
67.	1.	0.	-2.045263	-3.630818	-0.800396

S4 ^{1}H NMR Spectrum of eutypellenoid A (**1**) in $CDCl_3$.

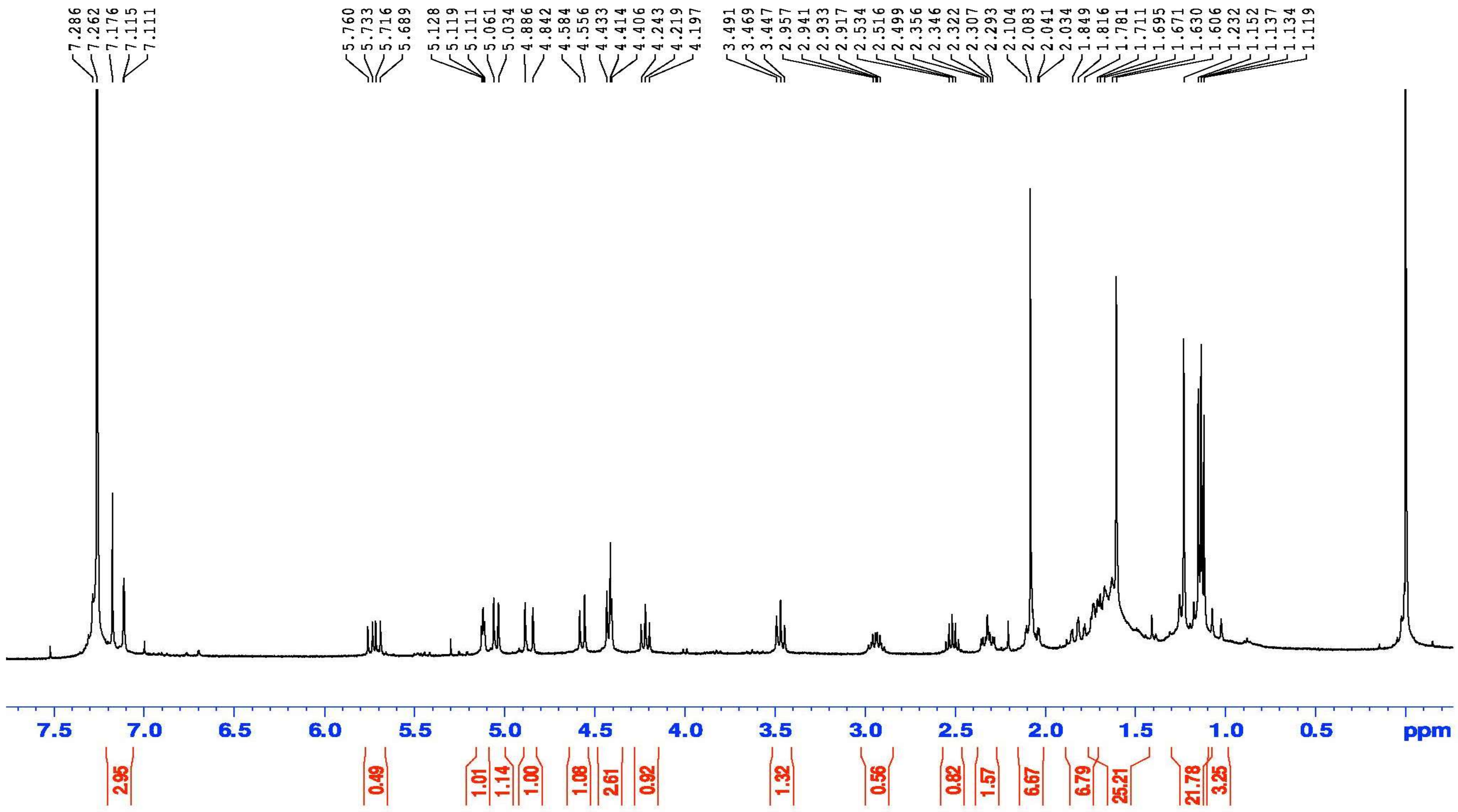

S5 ^{13}C NMR Spectrum of eutypellenoid A (**1**) in $CDCl_3$.

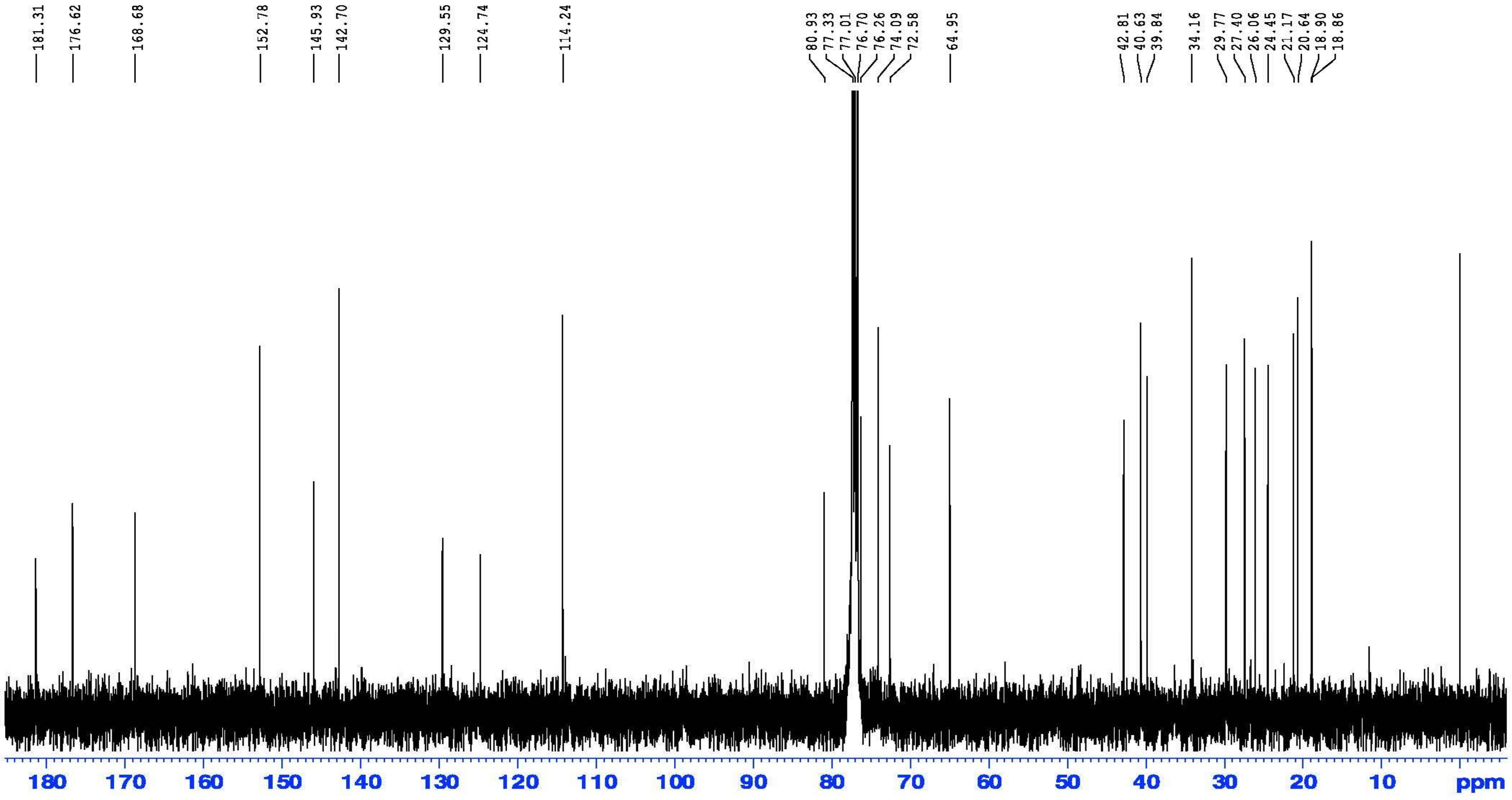

S6 DEPT135 Spectrum of eutypellenoid A (**1**) in $CDCl_3$.

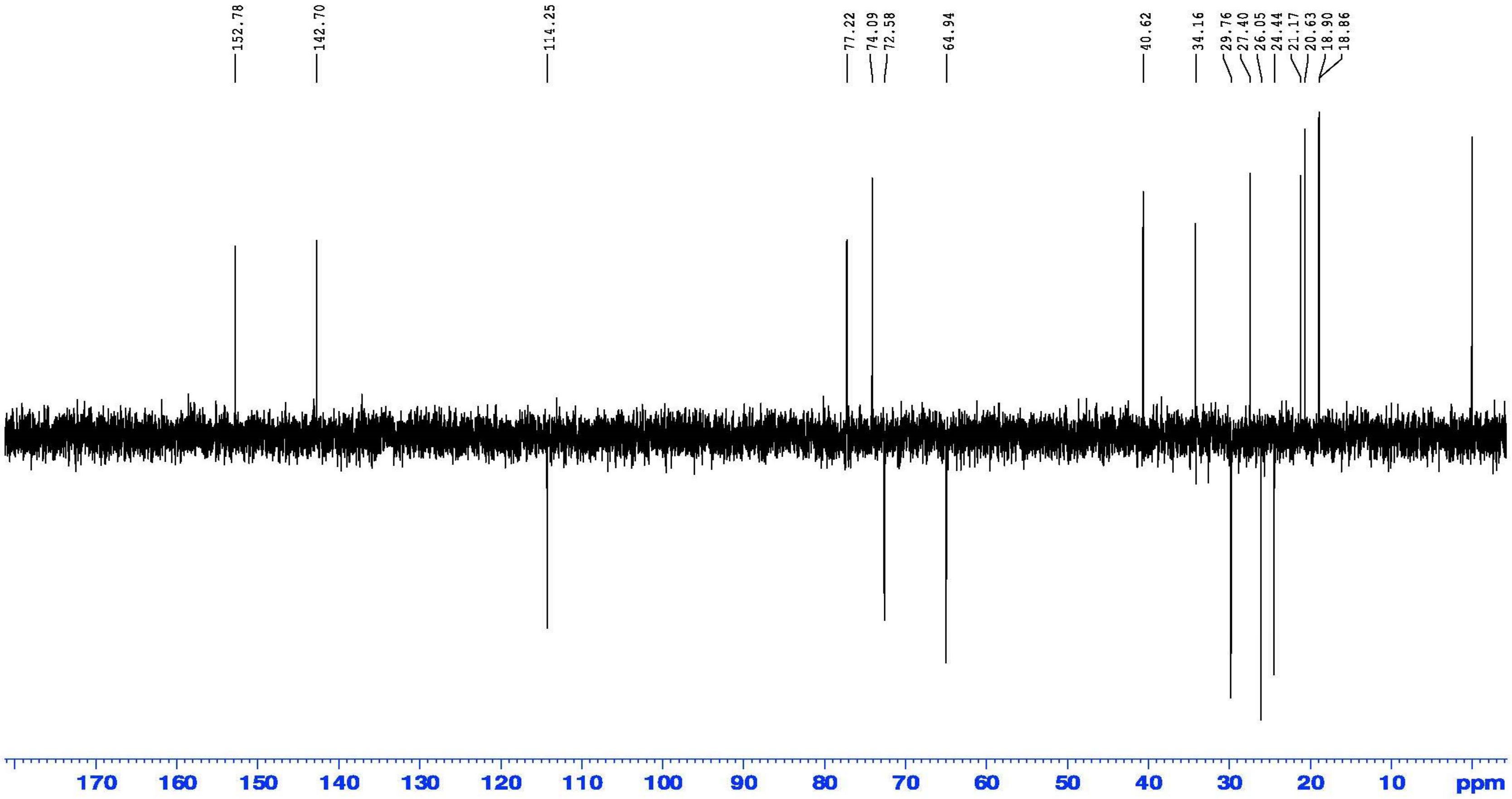

S7 HSQC Spectrum of eutypellenoid A (**1**) in $CDCl_3$.

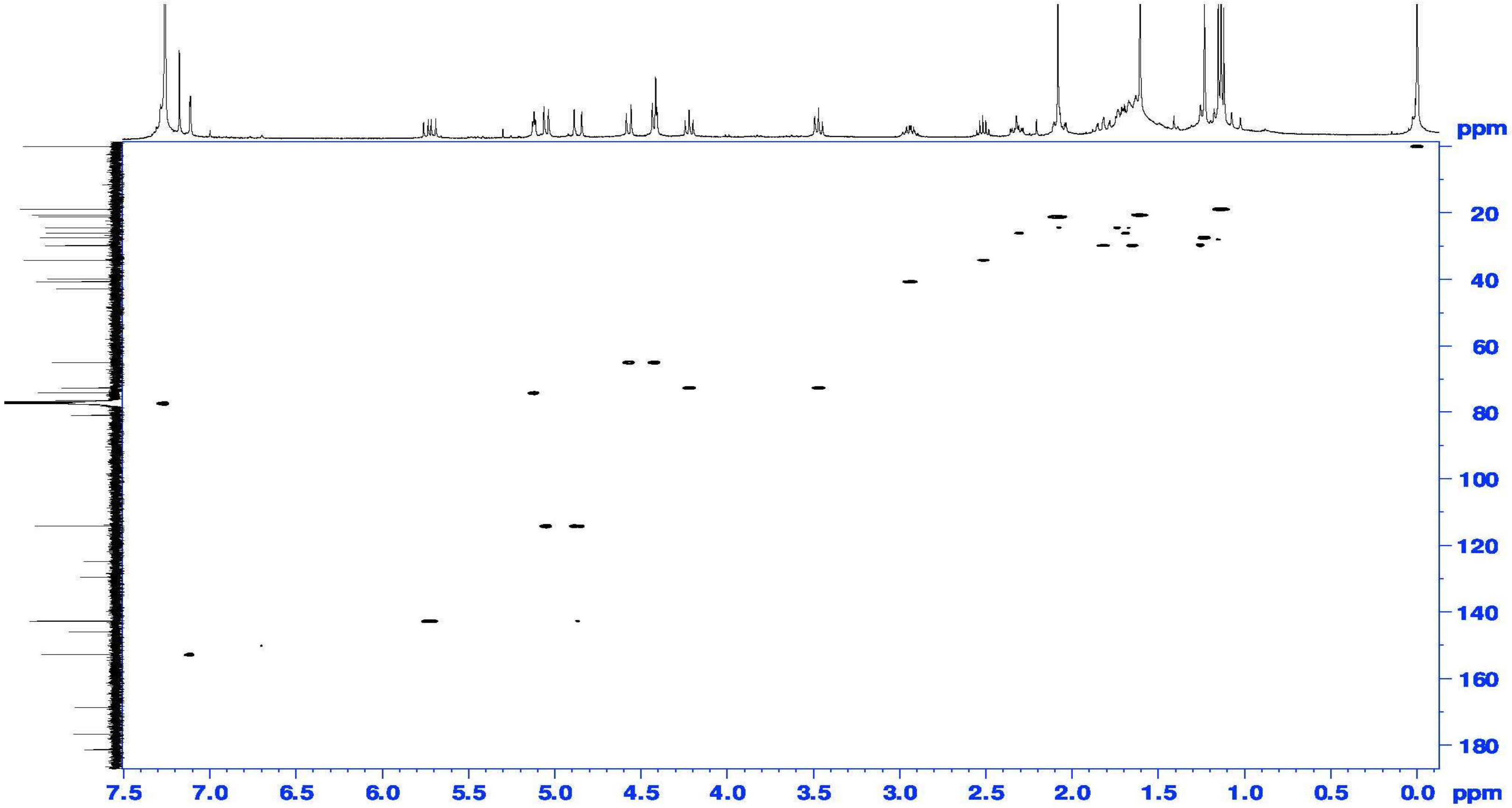

S8 COSY Spectrum of eutypellenoid A (**1**) in $CDCl_3$.

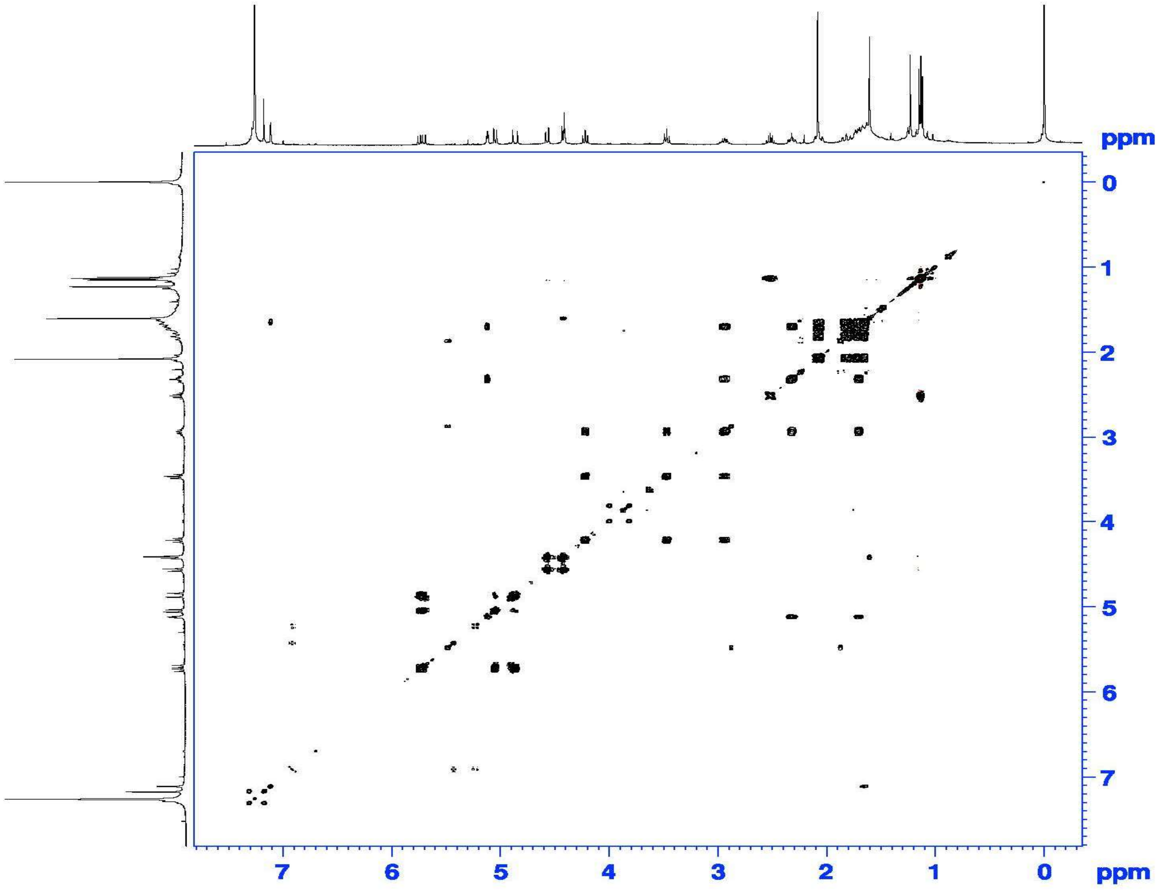

S9 HMBC Spectrum of eutypellenoid A (**1**) in $CDCl_3$.

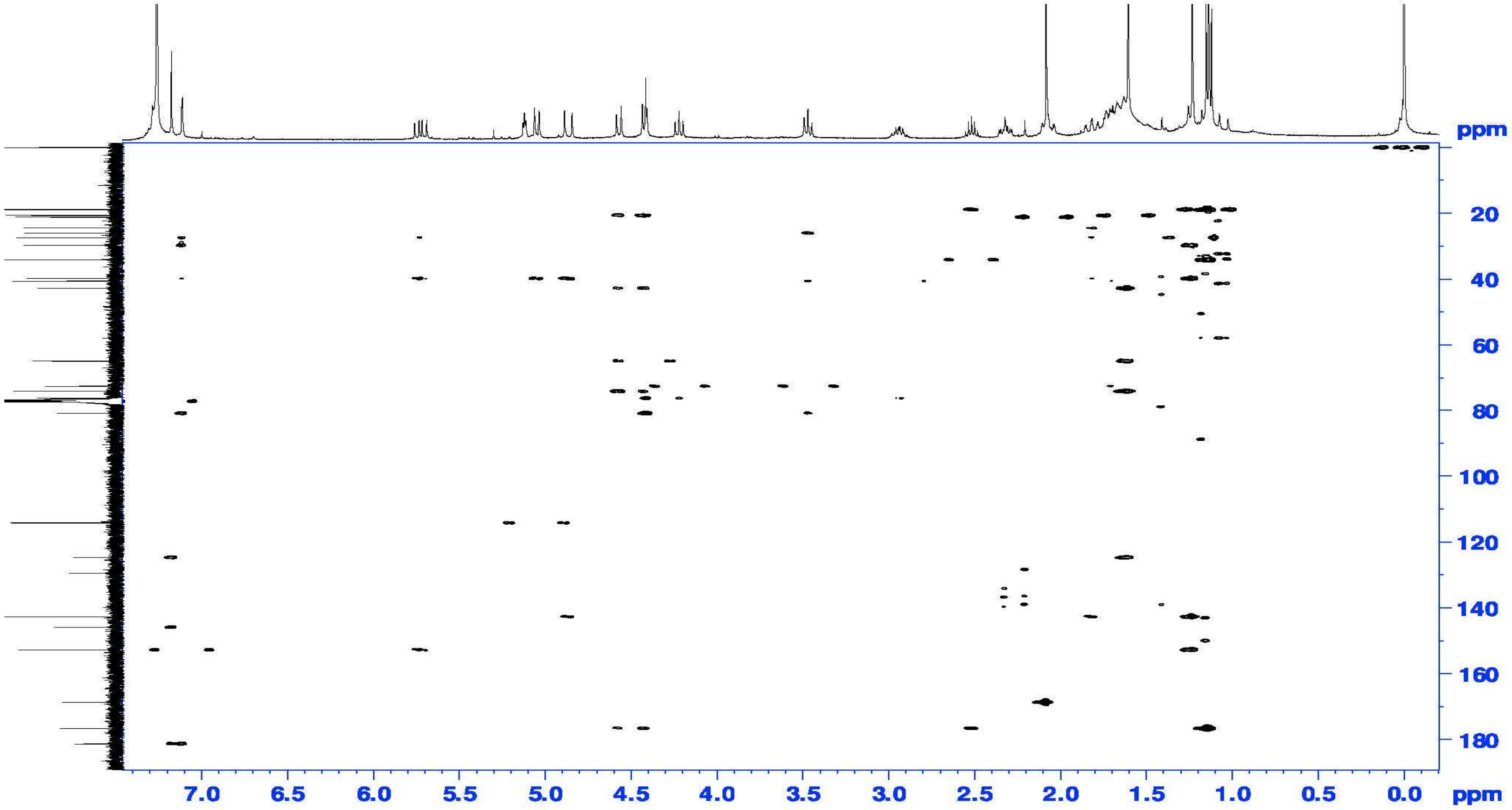

S10 NOESY Spectrum of eutypellenoid A (**1**) in $CDCl_3$.

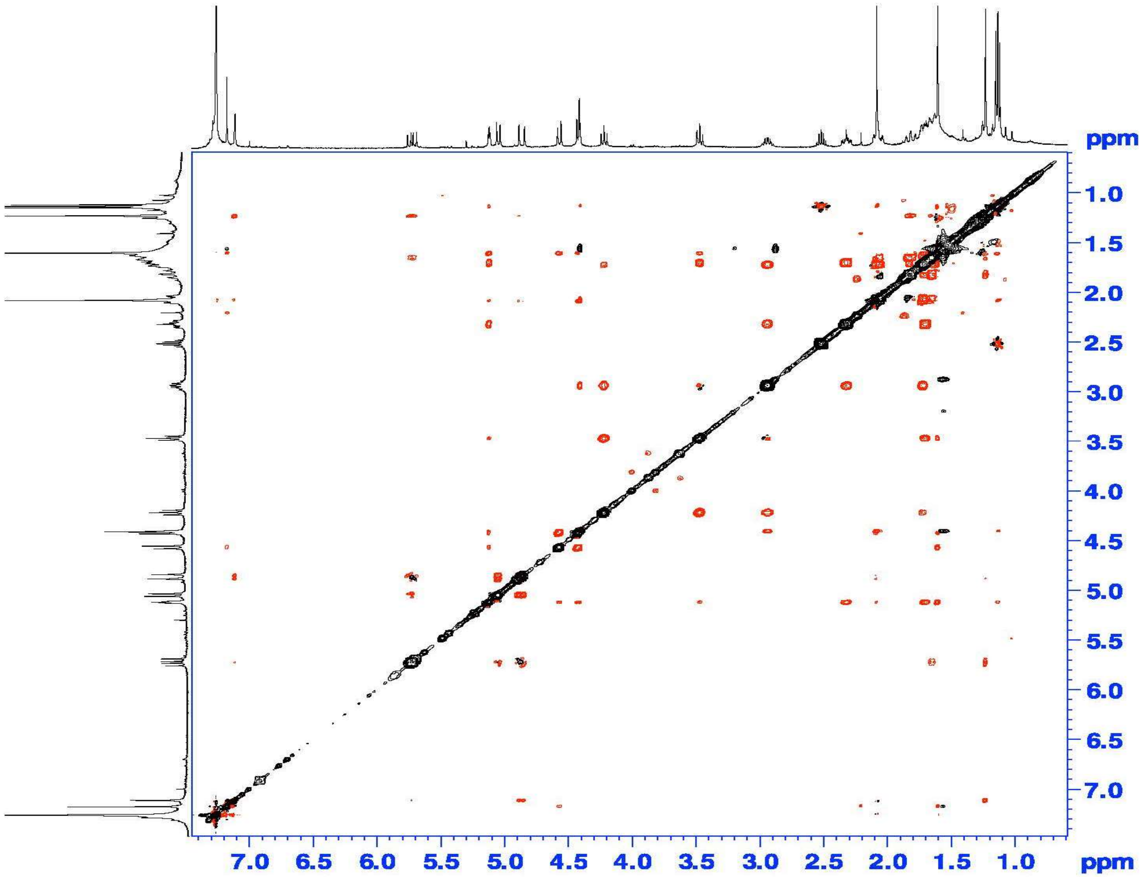

S11 HRESIMS of eutypellenoid A (**1**).

Qualitative Analysis Report

**Data Filename**	W-28-NEG.d	**Sample Name**	
**Sample Type**	Sample	**Position**	P2-E6
**Instrument Name**	Instrument 1	**User Name**	
**Acq Method**	SERUM-NEG-15MIN.m	**Acquired Time**	
**IRM Calibration Status**	Some Ions Missed	**DA Method**	Metabolomics-Default.m
**Comment**			

Sample Group **Info.**

User Spectra

Fragmentor Voltage	Collision Energy	Ionization Mode
120	0	ESI

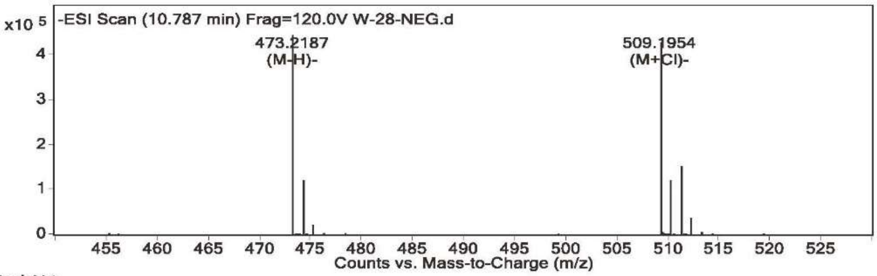

Peak List

m/z	z	Abund	Formula	Ion
473.2187	1	443433.6	C26 H33 O8	(M-H)-
474.2223	1	121615	C26 H33 O8	(M-H)-
509.1954	1	427242.4	C26 H34 Cl O8	(M+Cl)-
510.1991	1	120884.9	C26 H34 Cl O8	(M+Cl)-
511.1941	1	153480.1	C26 H34 Cl O8	(M+Cl)-

Formula Calculator Element Limits

Element	Min	Max
C	0	200
H	0	200
O	0	35

Formula Calculator Results

Formula	Best	Measured Mass	Tgt Mass	Diff (ppm)	Ion Species	Score
C26 H33 O8	TRUE	473.2187	473.2193	1.24	C26 H33 O8	98.78

--- End Of Report ---

S12 UV spectrum of eutypellenoid A (**1**).

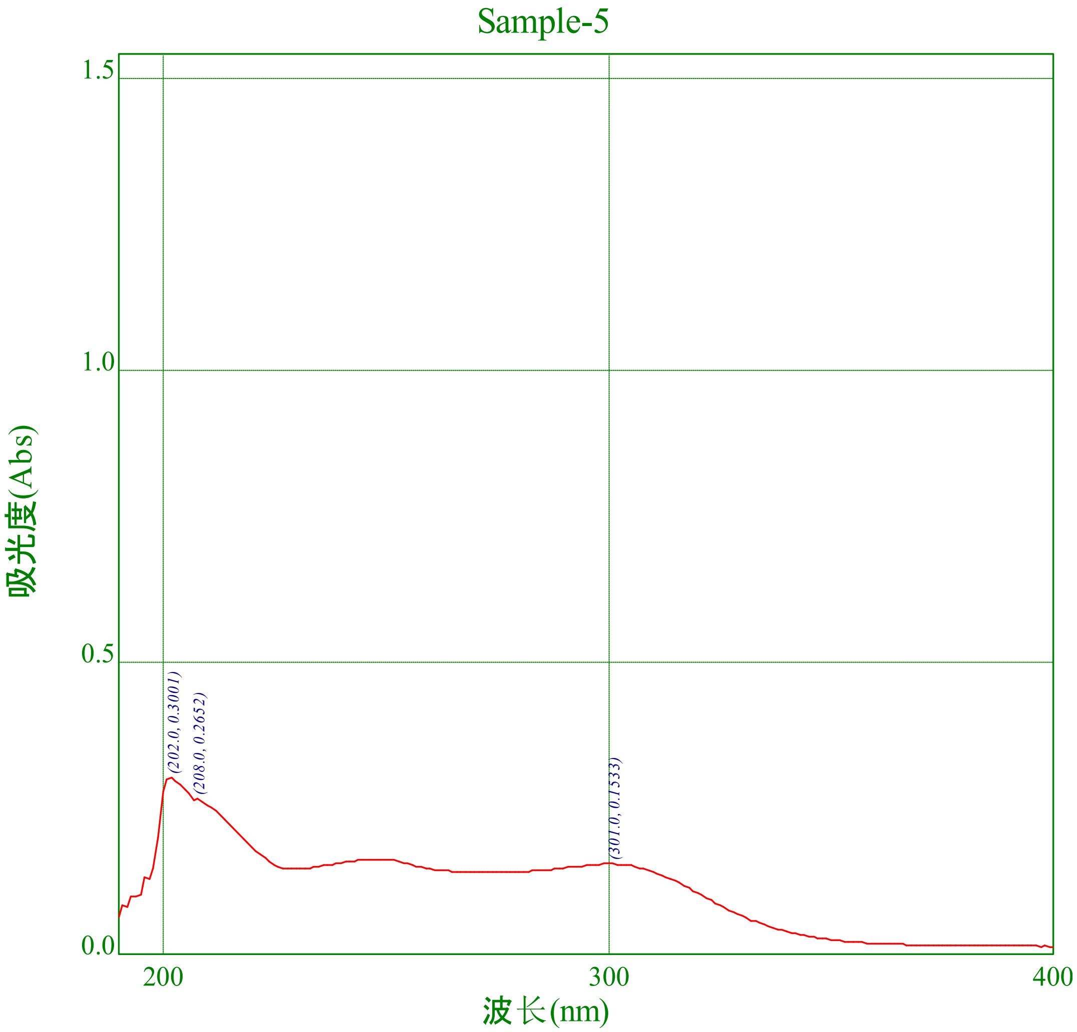

S13 IR spectrum of eutypellenoid A (**1**).

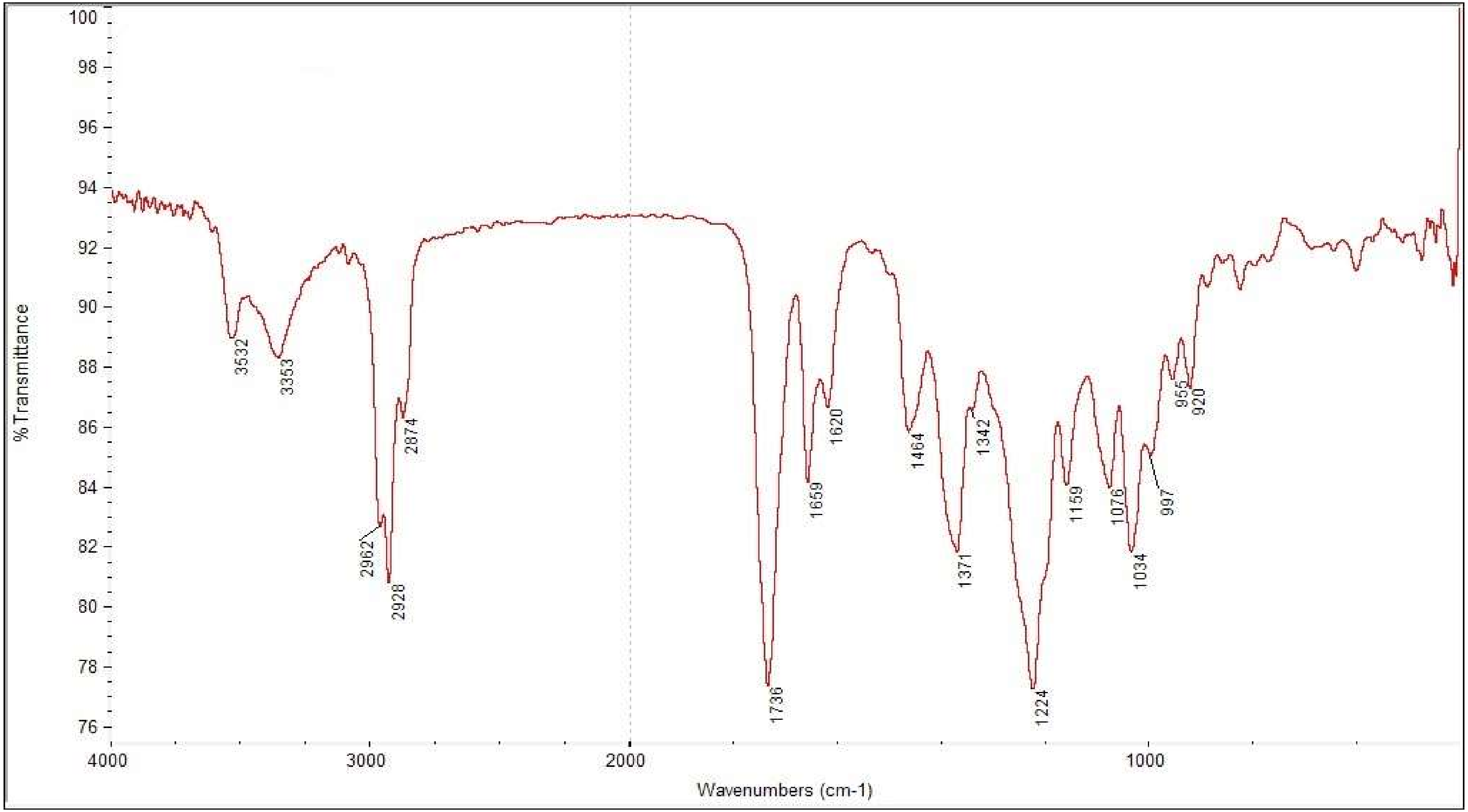

S14 ^{1}H NMR Spectrum of eutypellenoid B (**2**) in $CDCl_3$.

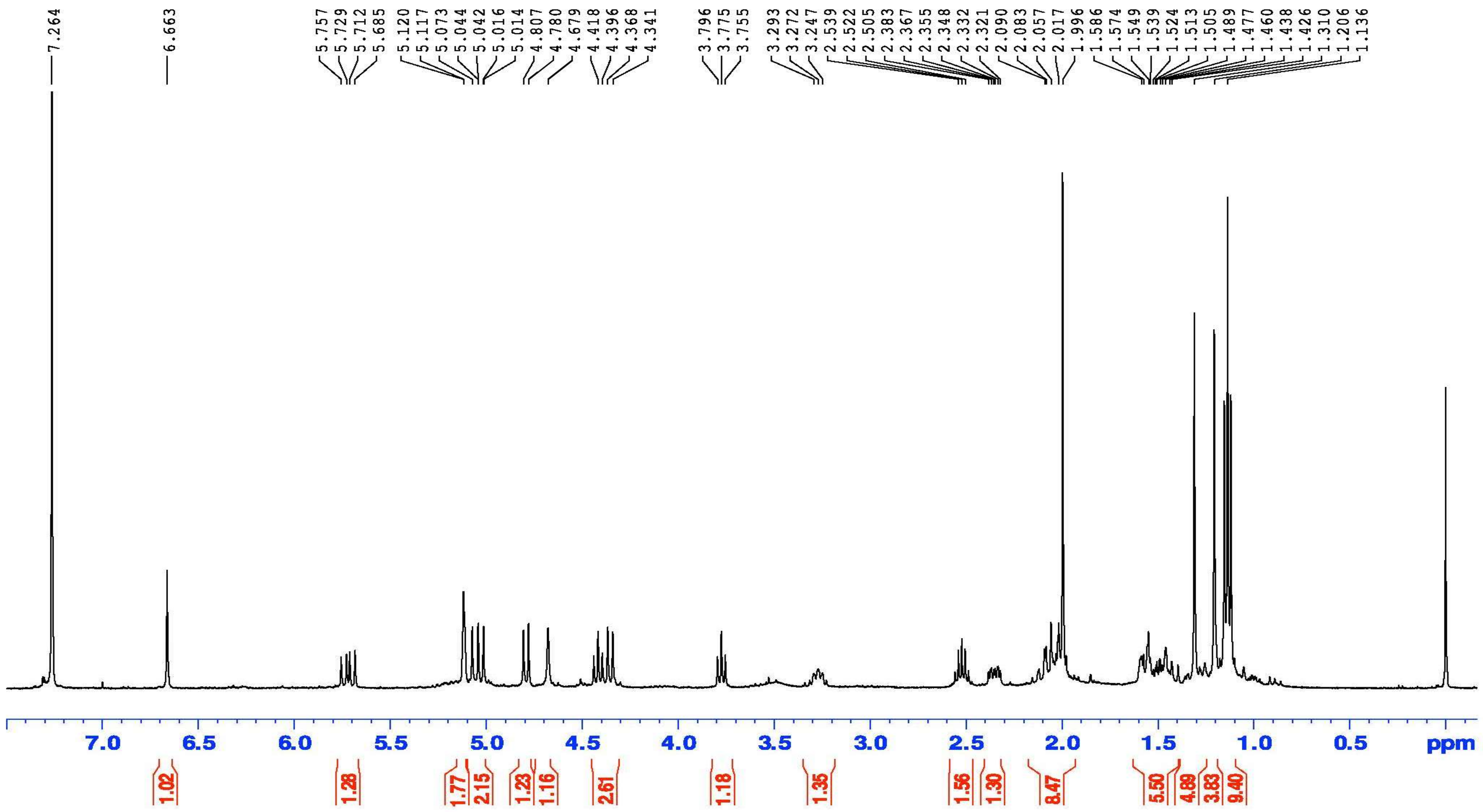

S15 ^{13}C NMR Spectrum of eutypellenoid B (**2**) in $CDCl_3$.

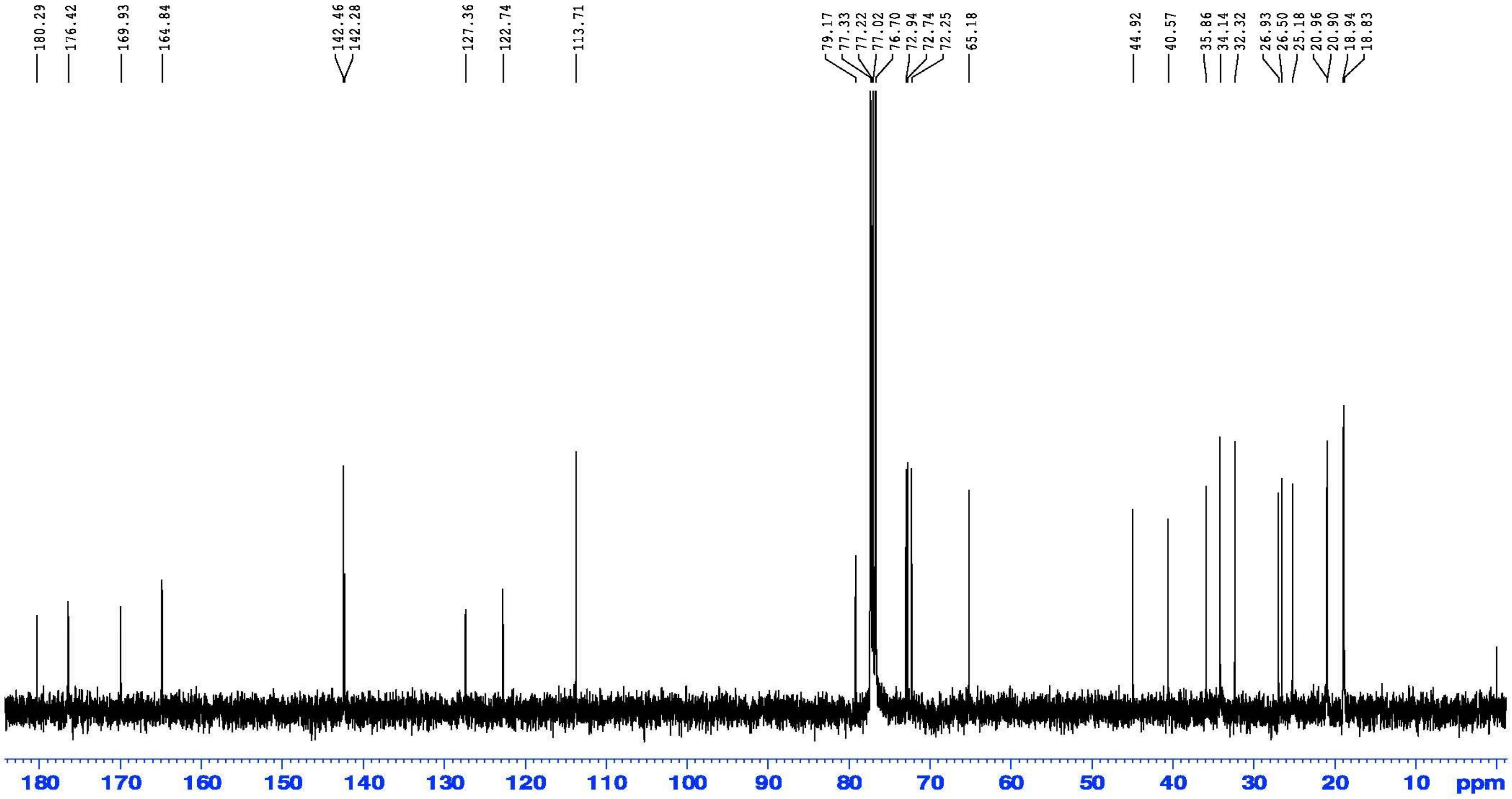

S16 DEPT135 Spectrum of eutypellenoid B (**2**) in $CDCl_3$.

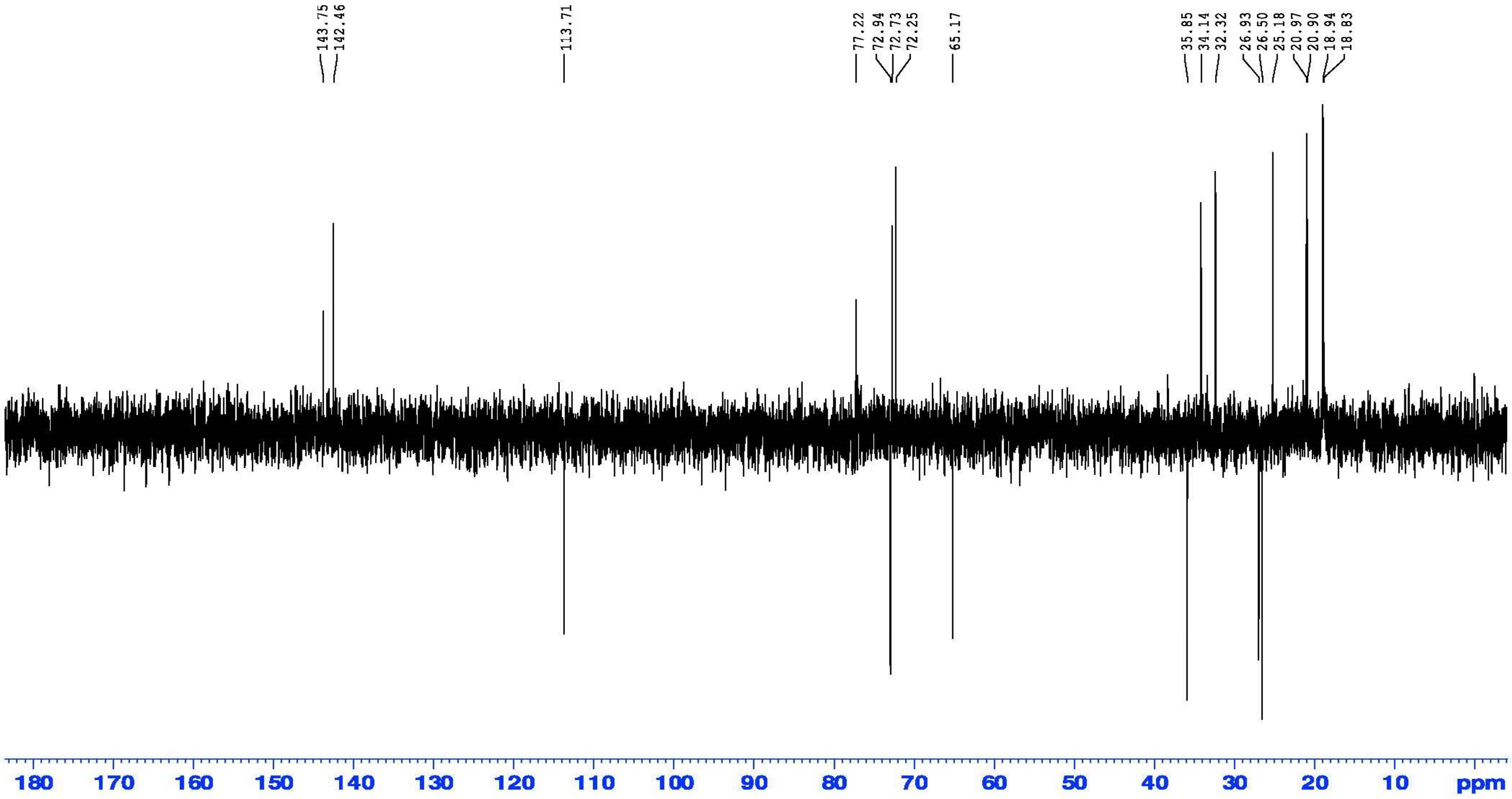

S17 HSQC Spectrum of eutypellenoid B (**2**) in $CDCl_3$.

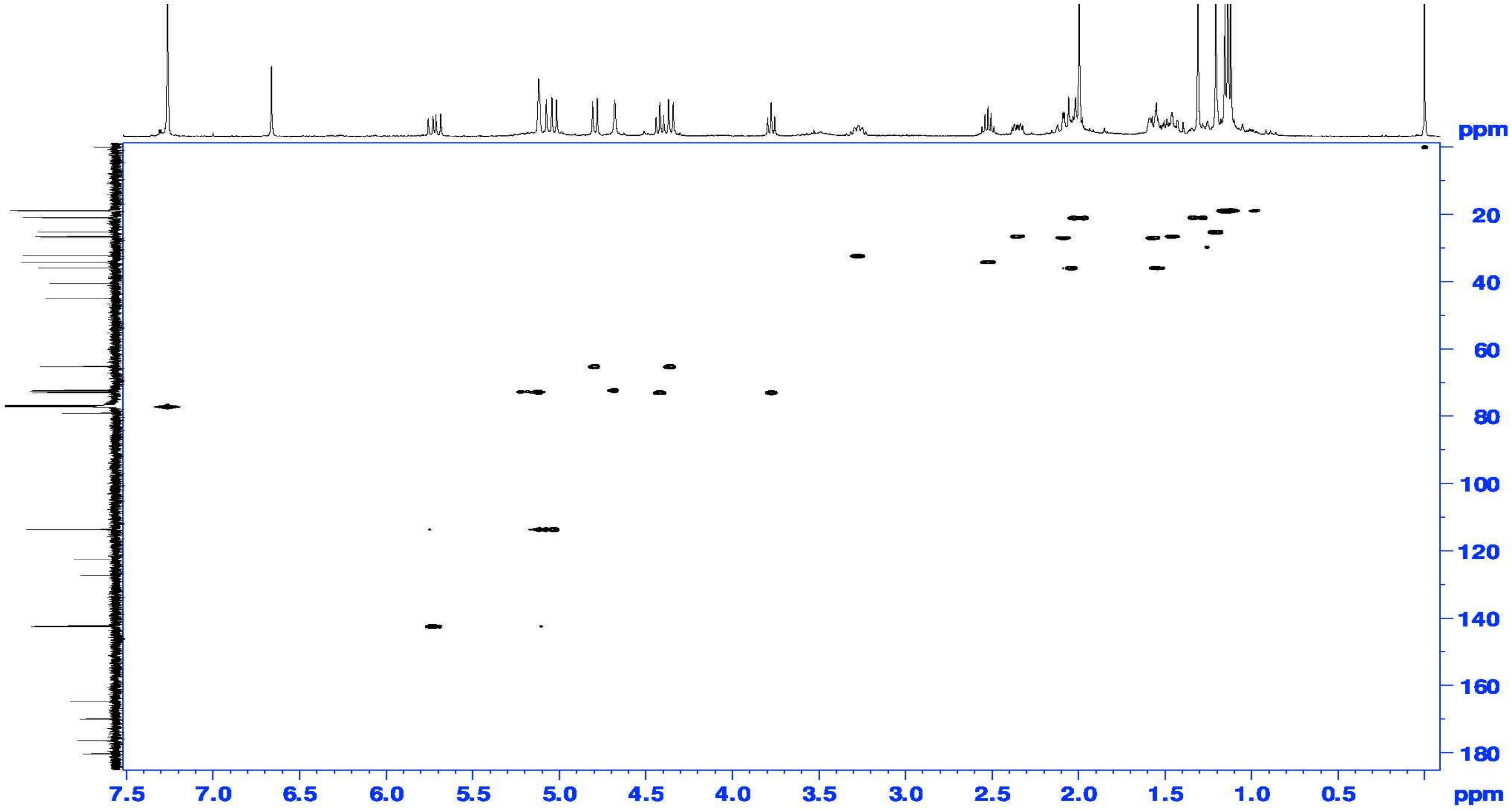

S18 COSY Spectrum of eutypellenoid B (**2**) in $CDCl_3$.

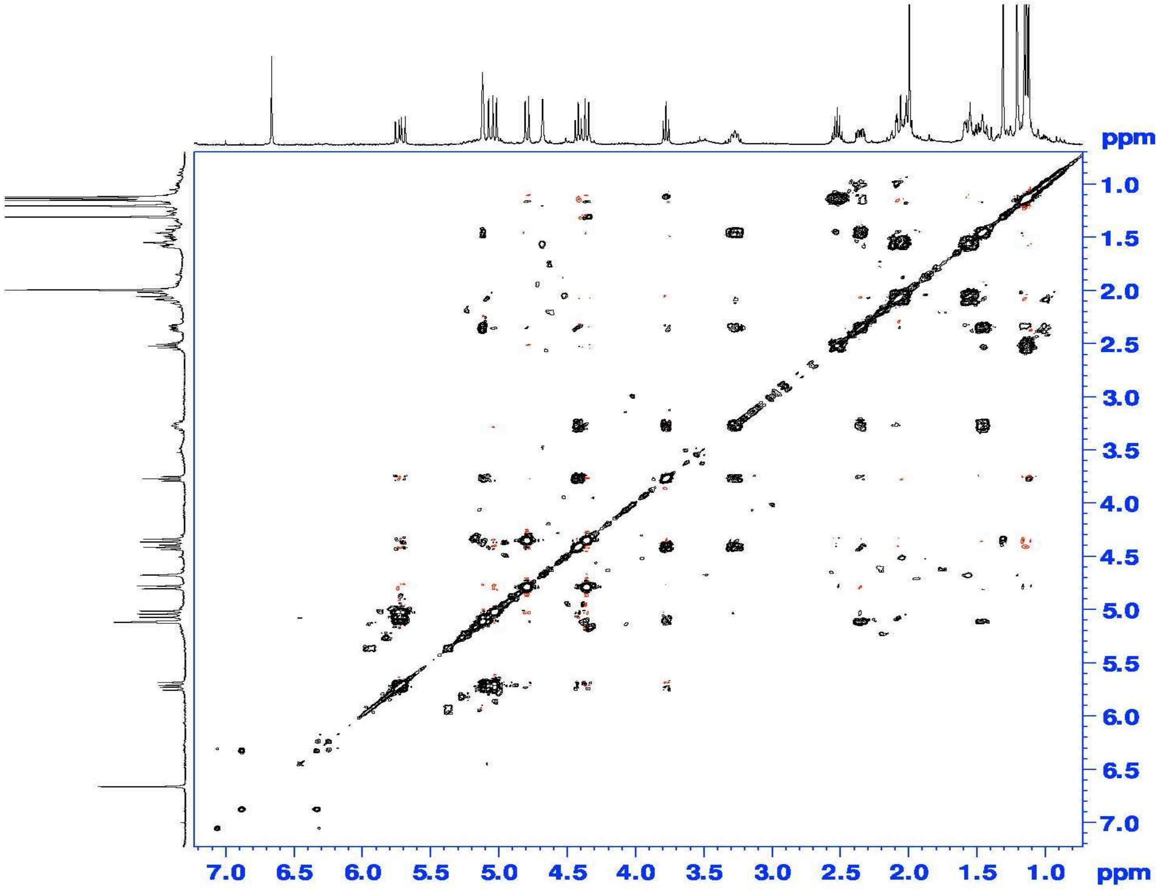

S19 HMBC Spectrum of eutypellenoid B (**2**) in $CDCl_3$.

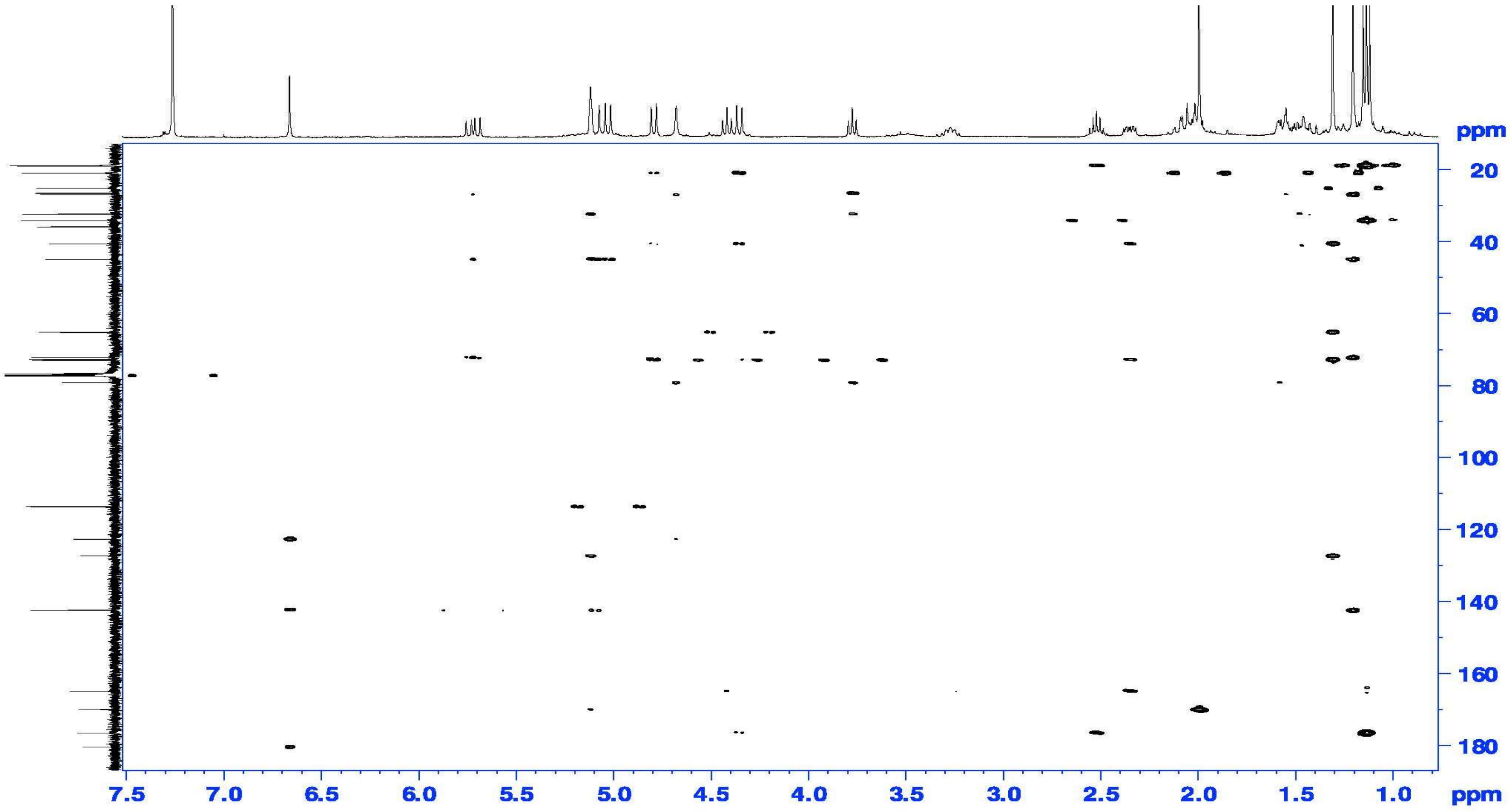

S20 NOESY Spectrum of eutypellenoid B (**2**) in $CDCl_3$.

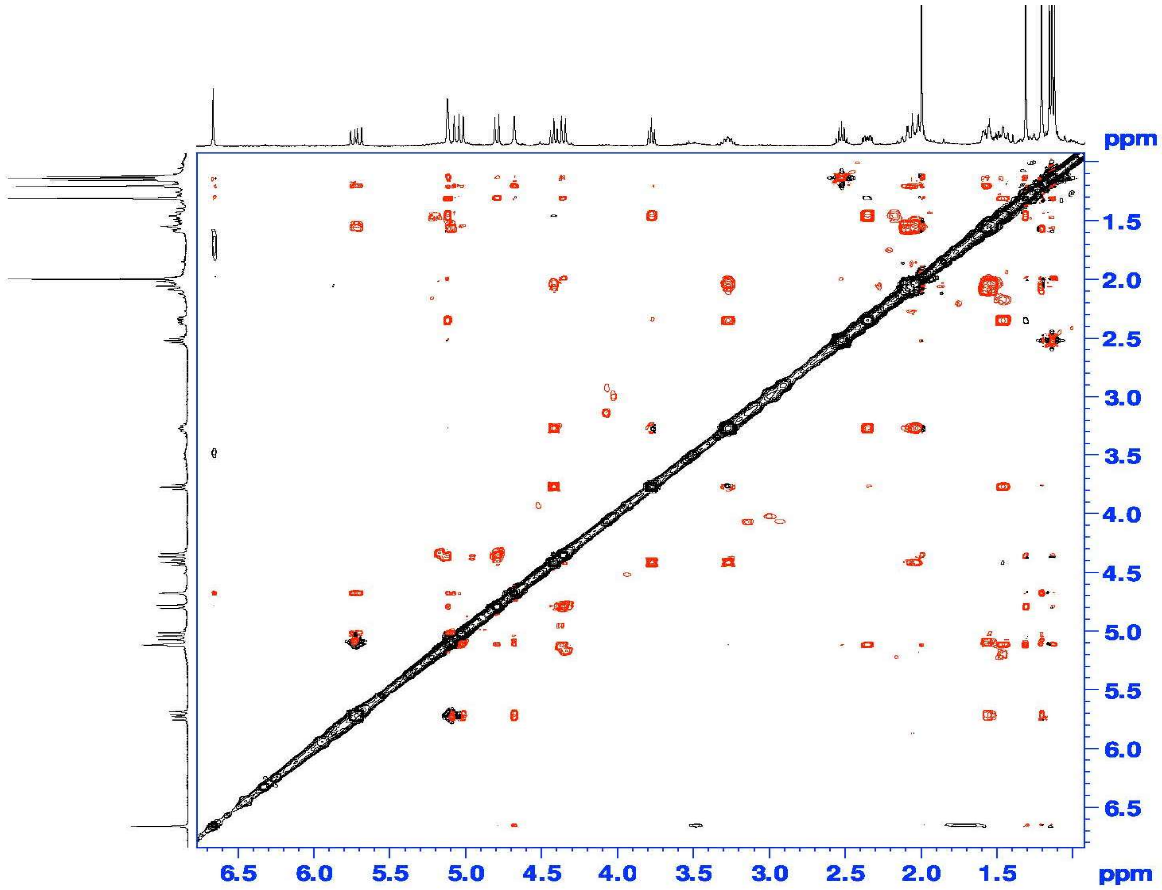

S21 HRESIMS of eutypellenoid B (**2**).

State Key Laboratory of Organometallic Chemistry
Shanghai Institute of Organic Chemistry
Chinese Academy of Sciences
ESI High Resolution MS Date Report

Data Filename	W-22.d
Sample Name	W-22
User Name	
Acquired Time	4/18/2018 2:59:06 PM
Instrument	

Agilent Technologies 6224 TOF LC/MS

User Spectra

Fragmentor Voltage	Collision Energy	Ionization Mode
150	0	ESI

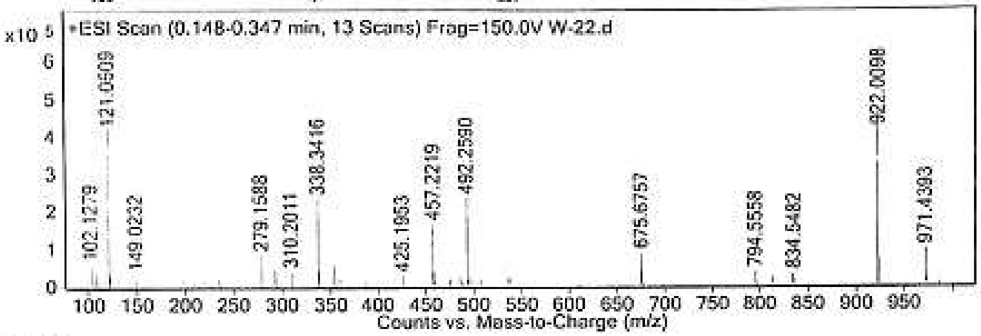

Peak List

m/z	z	Abund	Formula	Ion
121.0509		584318.8		
279.1588		84981.3		
338.3416	1	233405		
355.3678		61097		
457.2219	1	166997.7		
492.259	1	237611.4	C26 H38 N O8	(M+NH4)+
675.6757	1	89711.5		
922.0098	1	464332.4		
923.0127	1	71438.5		
971.4393	1	97784.1		

Formula Calculator Results

IonFormula	Measured Mass	Tgt Mass	Diff (ppm)	Score
C26 H38 N O8	492.259	492.2592	0.4	92.62
C24 H36 N4 O7	492.259	492.2579	-2.44	90.14

--- End Of Report ---

S22 UV spectrum of eutypellenoid B (**2**).

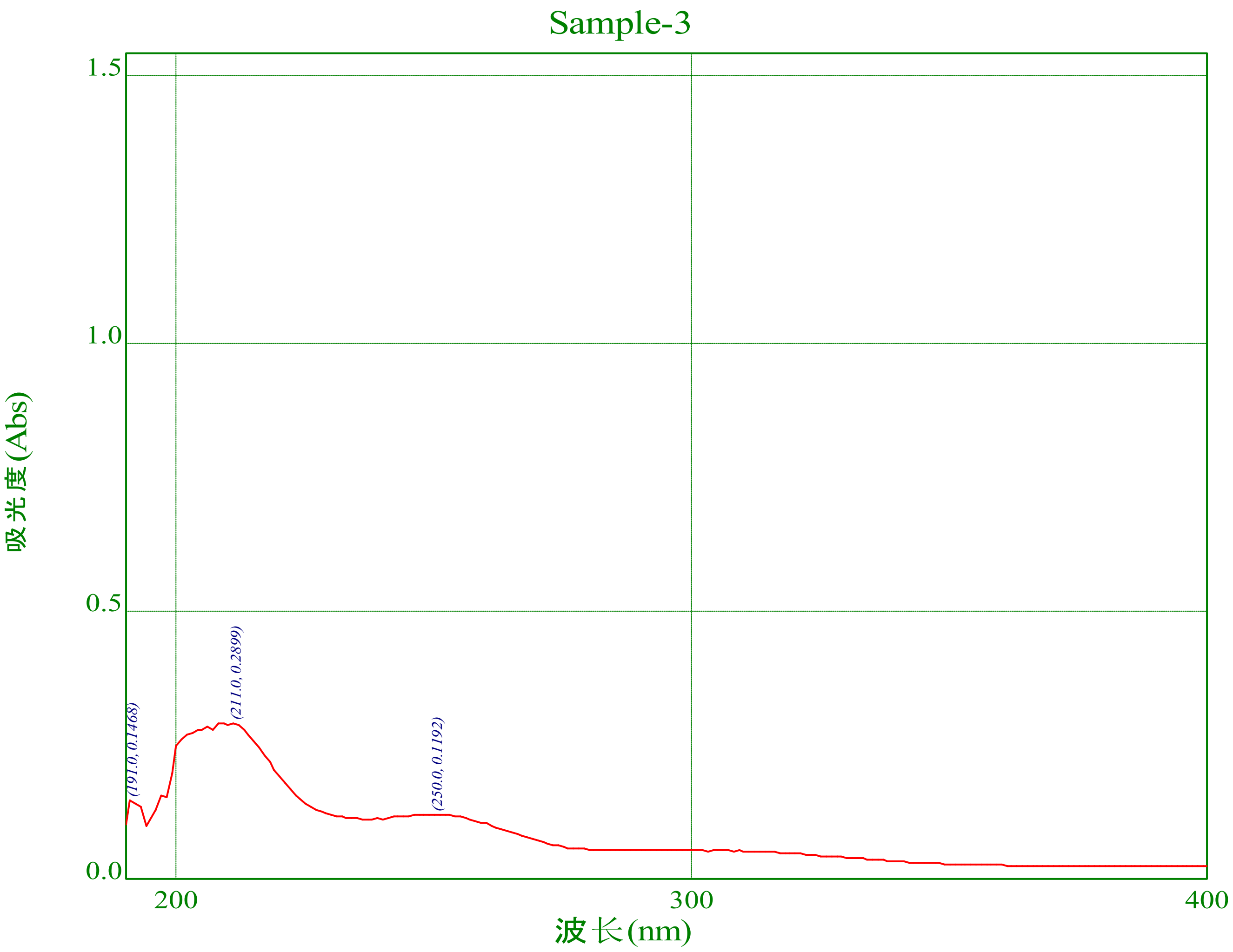

S23 IR spectrum of eutypellenoid B (**2**).

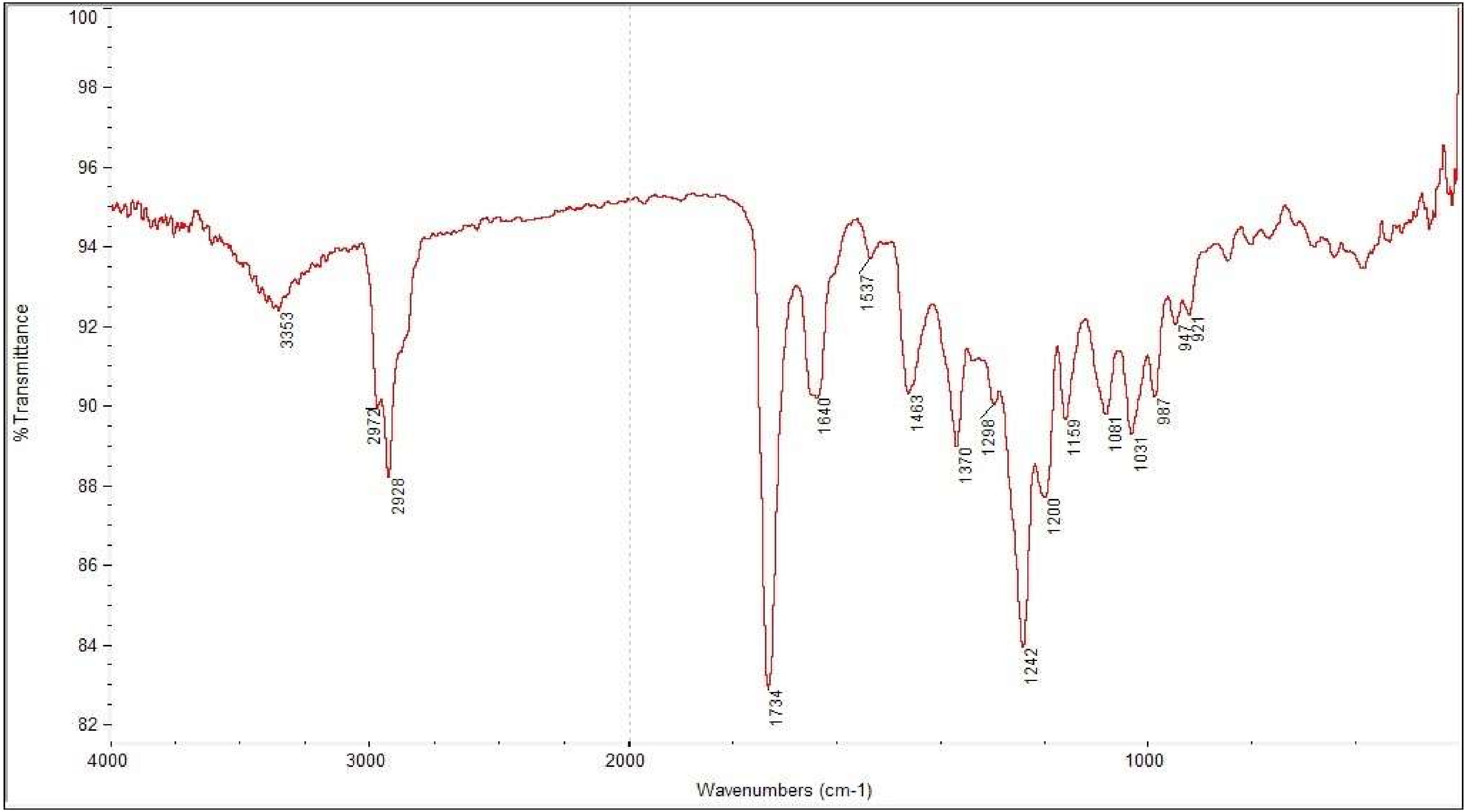

S24 ^{1}H NMR Spectrum of eutypellenoid C (**3**) in $CDCl_3$.

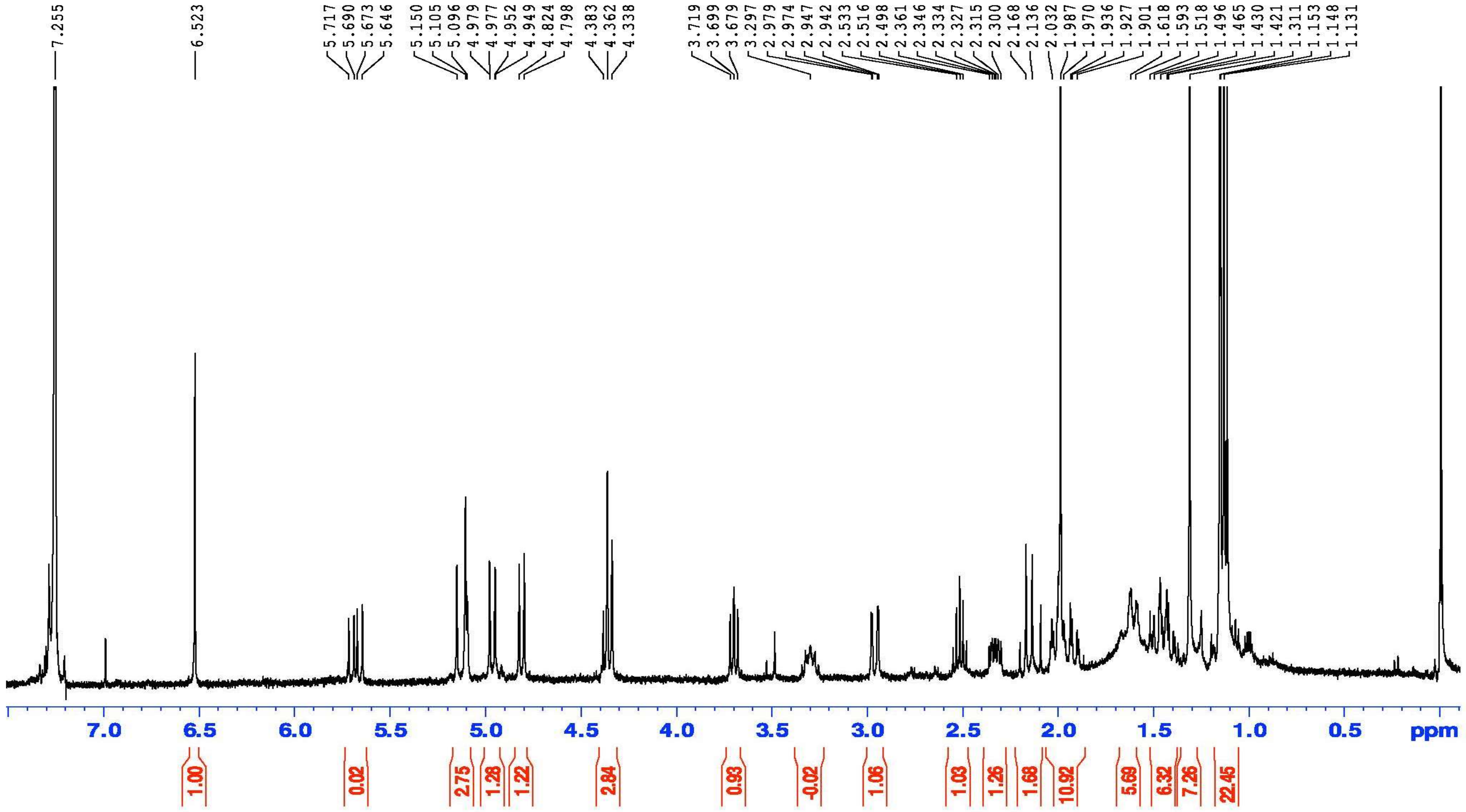

S25 ^{13}C NMR Spectrum of eutypellenoid C (**3**) in $CDCl_3$.

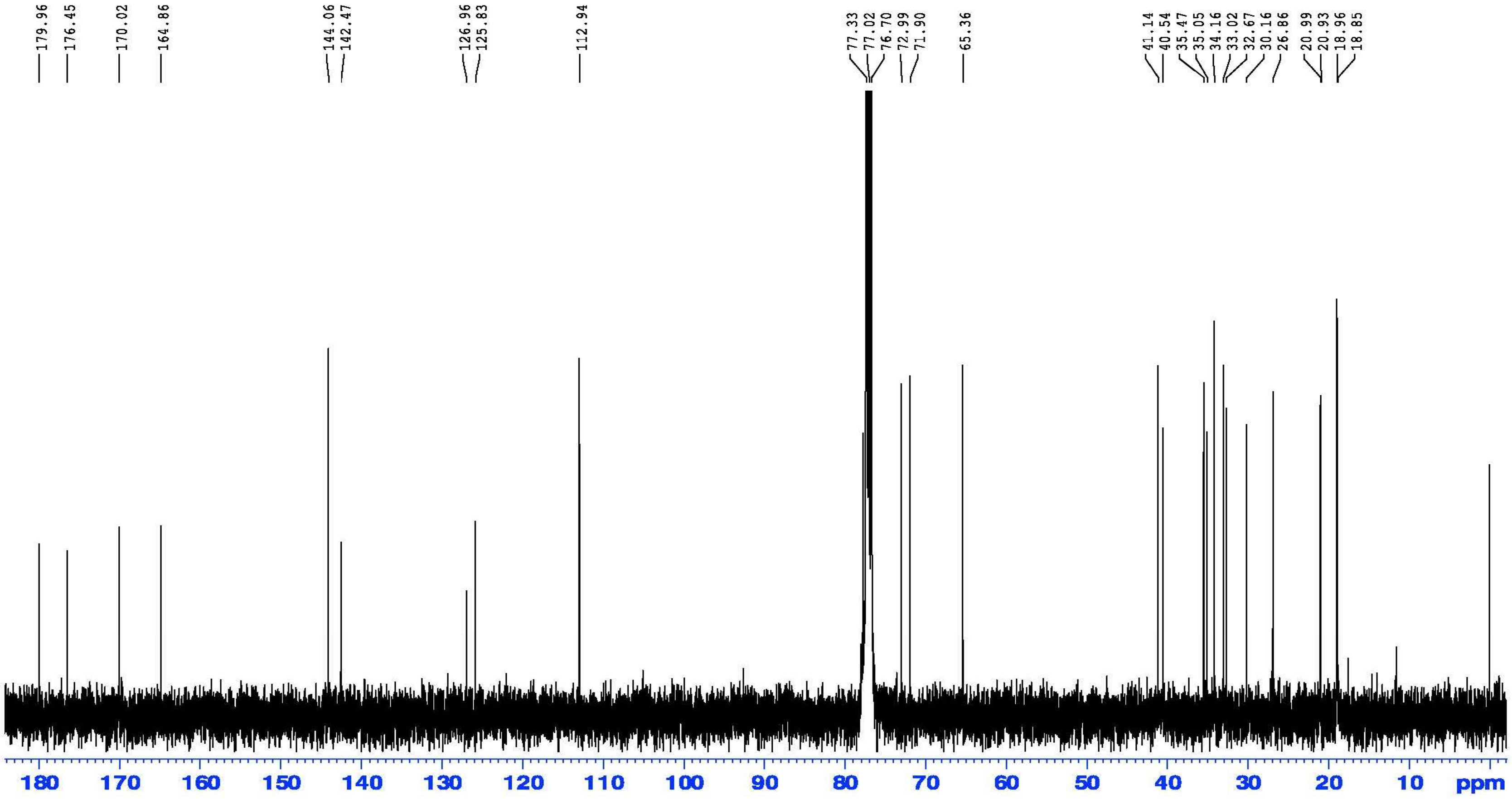

S26 DEPT135 Spectrum of eutypellenoid C (**3**) in $CDCl_3$.

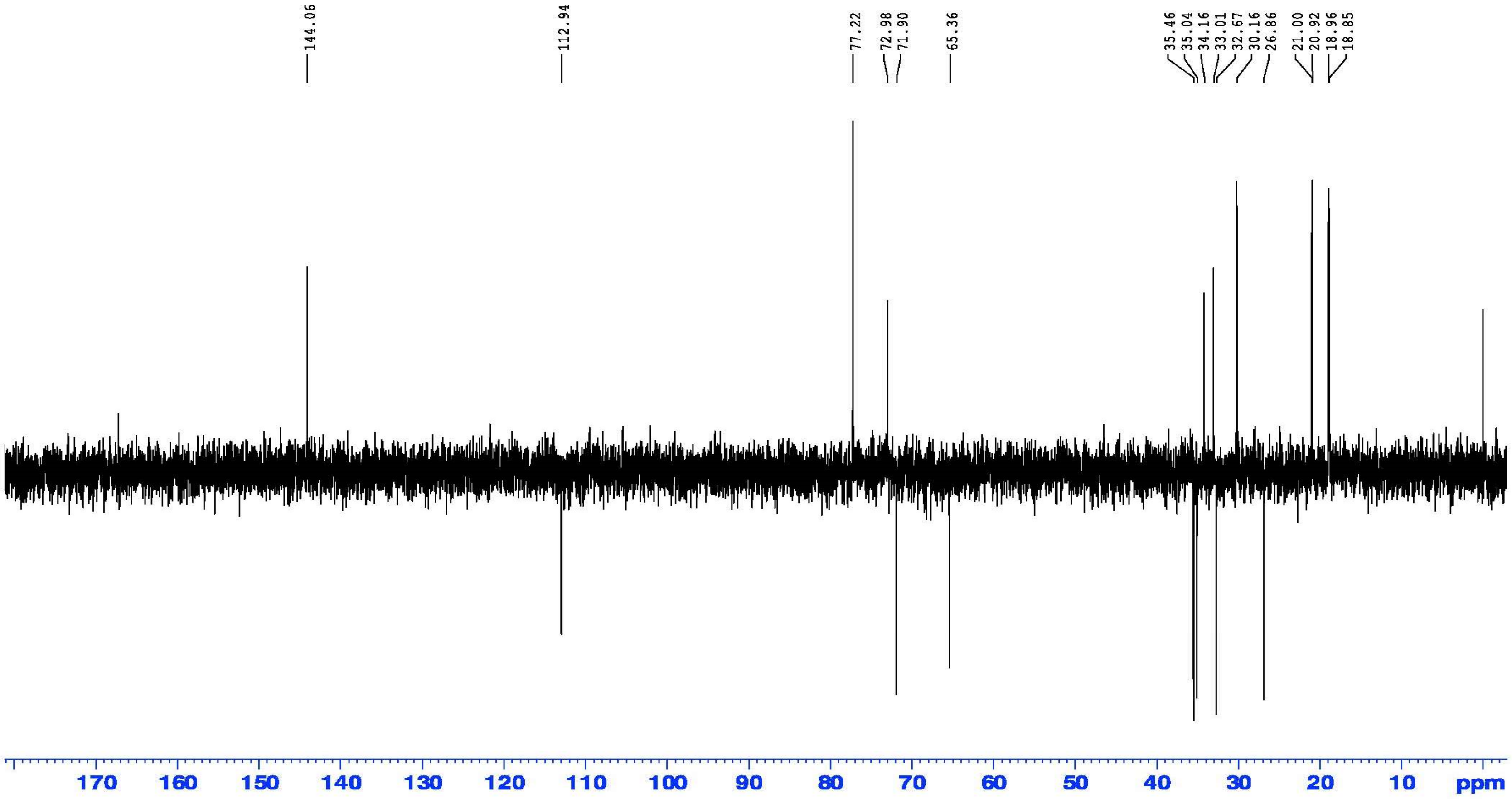

S27 HSQC Spectrum of eutypellenoid C (**3**) in $CDCl_3$.

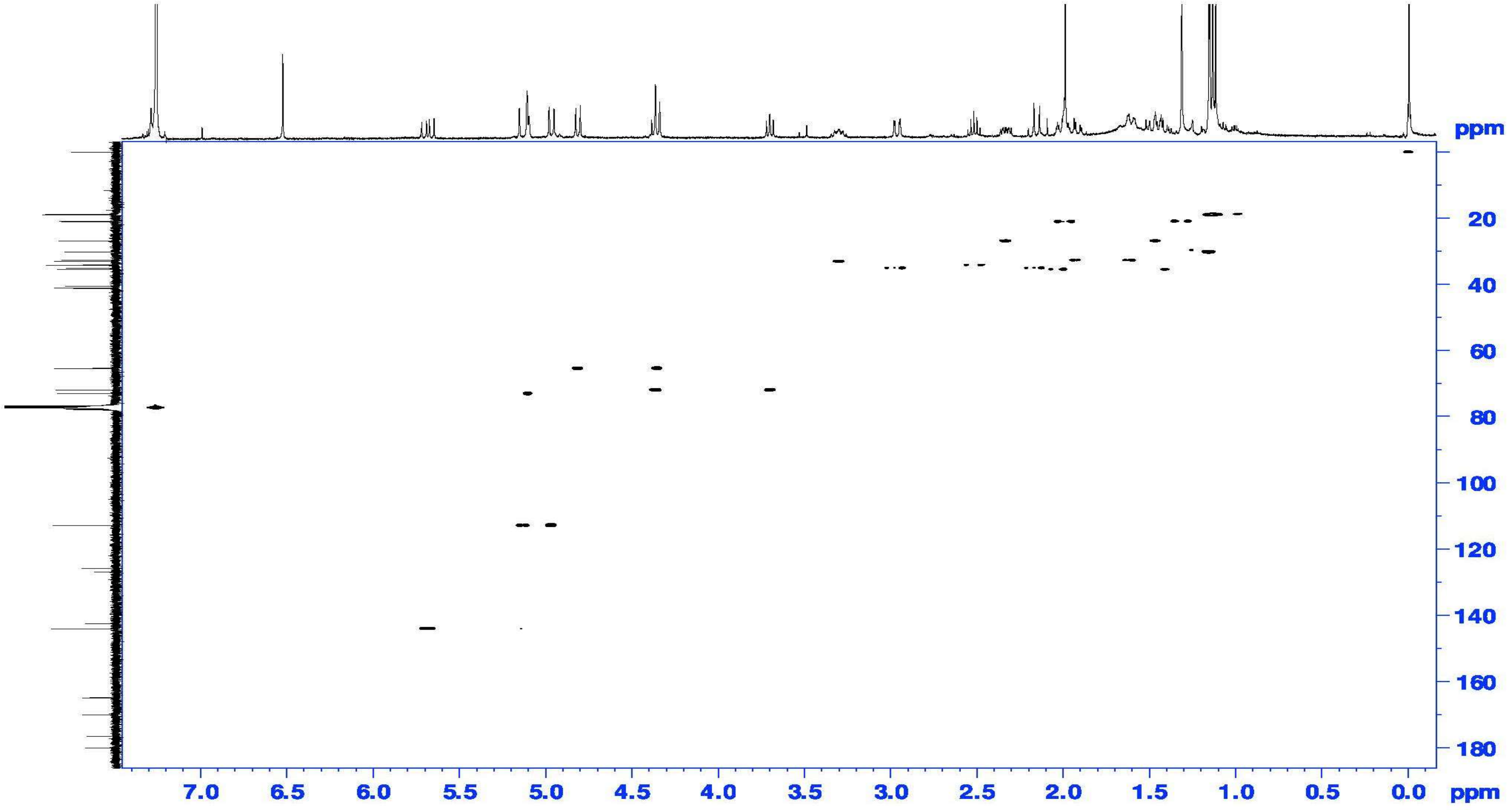

S28 COSY Spectrum of eutypellenoid C (**3**) in $CDCl_3$.

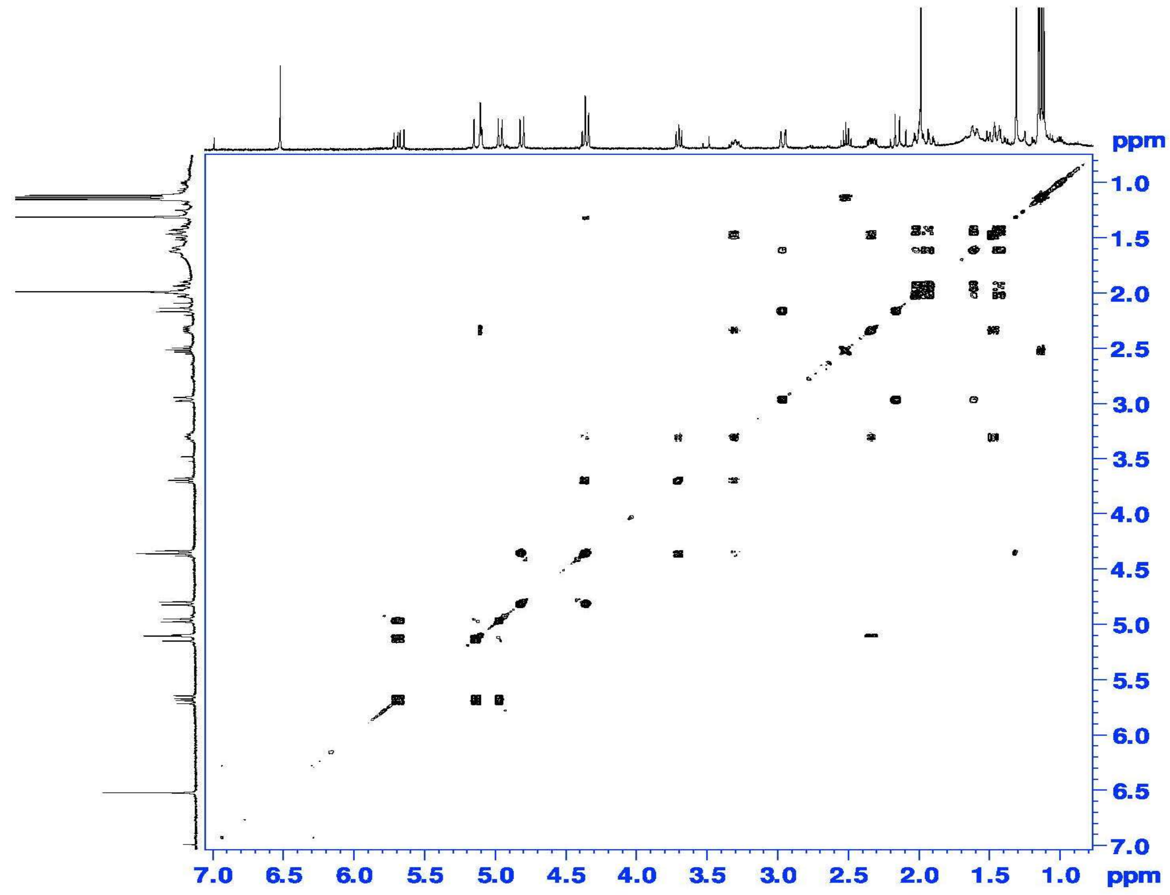

S29 HMBC Spectrum of eutypellenoid C (**3**) in $CDCl_3$.

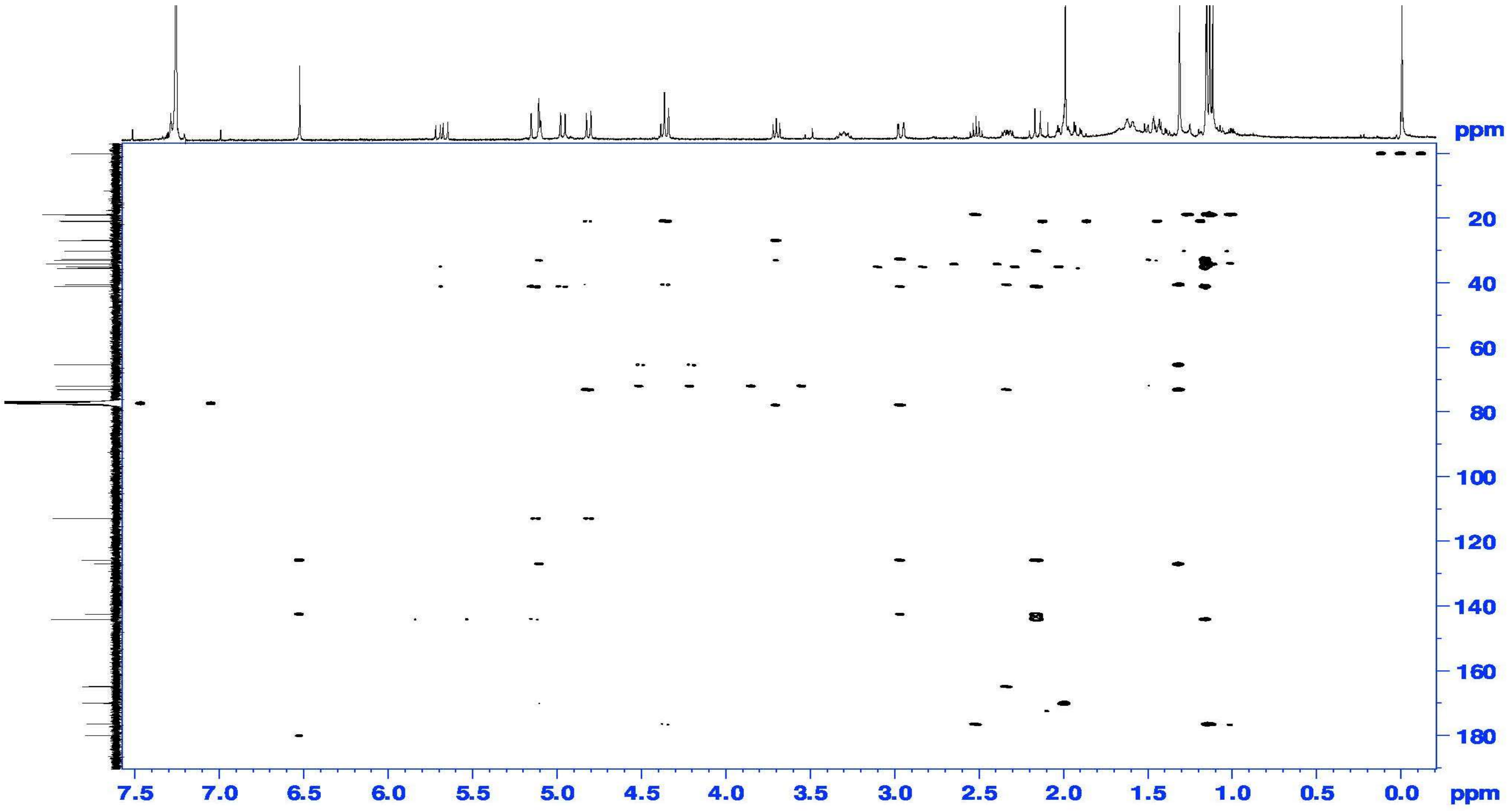

S30 NOESY Spectrum of eutypellenoid C (**3**) in $CDCl_3$.

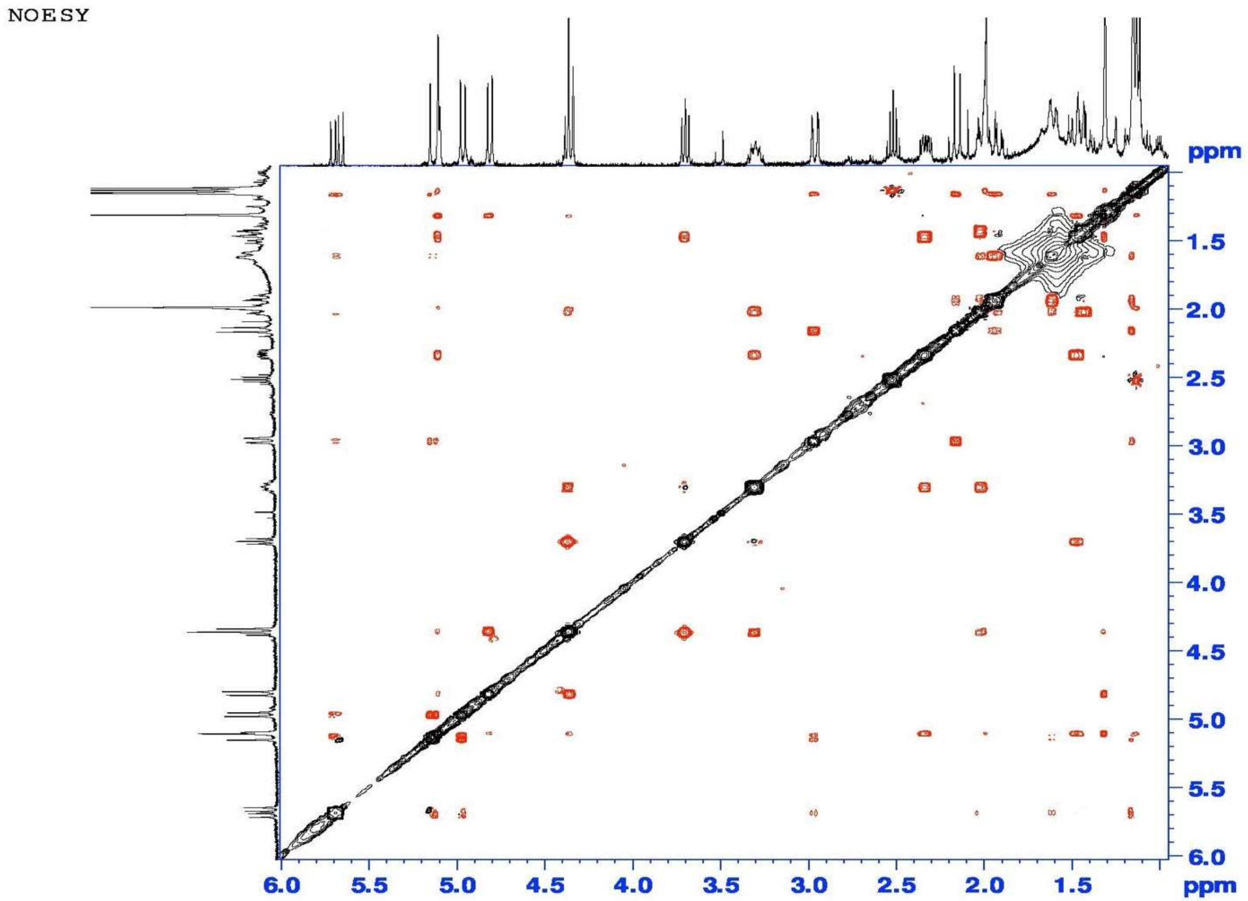

S31 HRESIMS of eutypellenoid C (**3**).

Qualitative Analysis Report

**Data Filename**	W-29-NEGd.d	**Sample Name**	
**Sample Type**	Sample	**Position**	P2-E7
**Instrument Name**	Instrument 1	**User Name**	
**Acq Method**	SERUM-NEG-15MIN.m	**Acquired Time**	
**IRM Calibration Status**	Some Ions Missed	**DA Method**	Metabolomics-Default.m
**Comment**			

Sample Group **Info.**

User Spectra

Fragmentor Voltage	Collision Energy	Ionization Mode
120	0	ESI

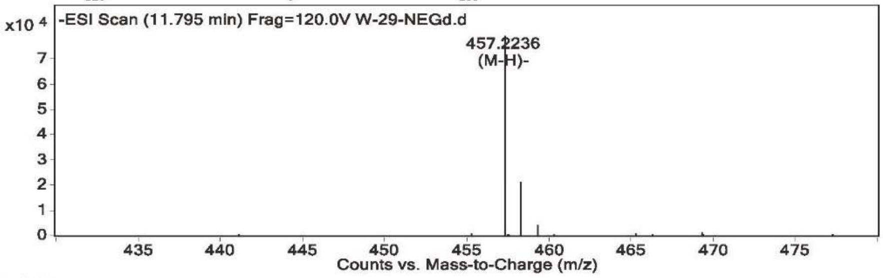

Peak List

m/z	Abund	Formula	Ion
457.2236	79483.5	C26 H33 O7	(M-H)-
804.5756	23846.4		
885.5491	30619.2		

Formula Calculator Element Limits

Element	Min	Max
C	0	200
H	0	200
O	0	35

Formula Calculator Results

Formula	Best	Measured Mass	Tgt Mass	Diff (ppm)	Ion Species	Score
C26 H33 O7	TRUE	457.2236	457.2232	-0.99	C26 H33 O7	98.49

--- End Of Report ---

S32 UV spectrum of eutypellenoid C (**3**) in MeOH.

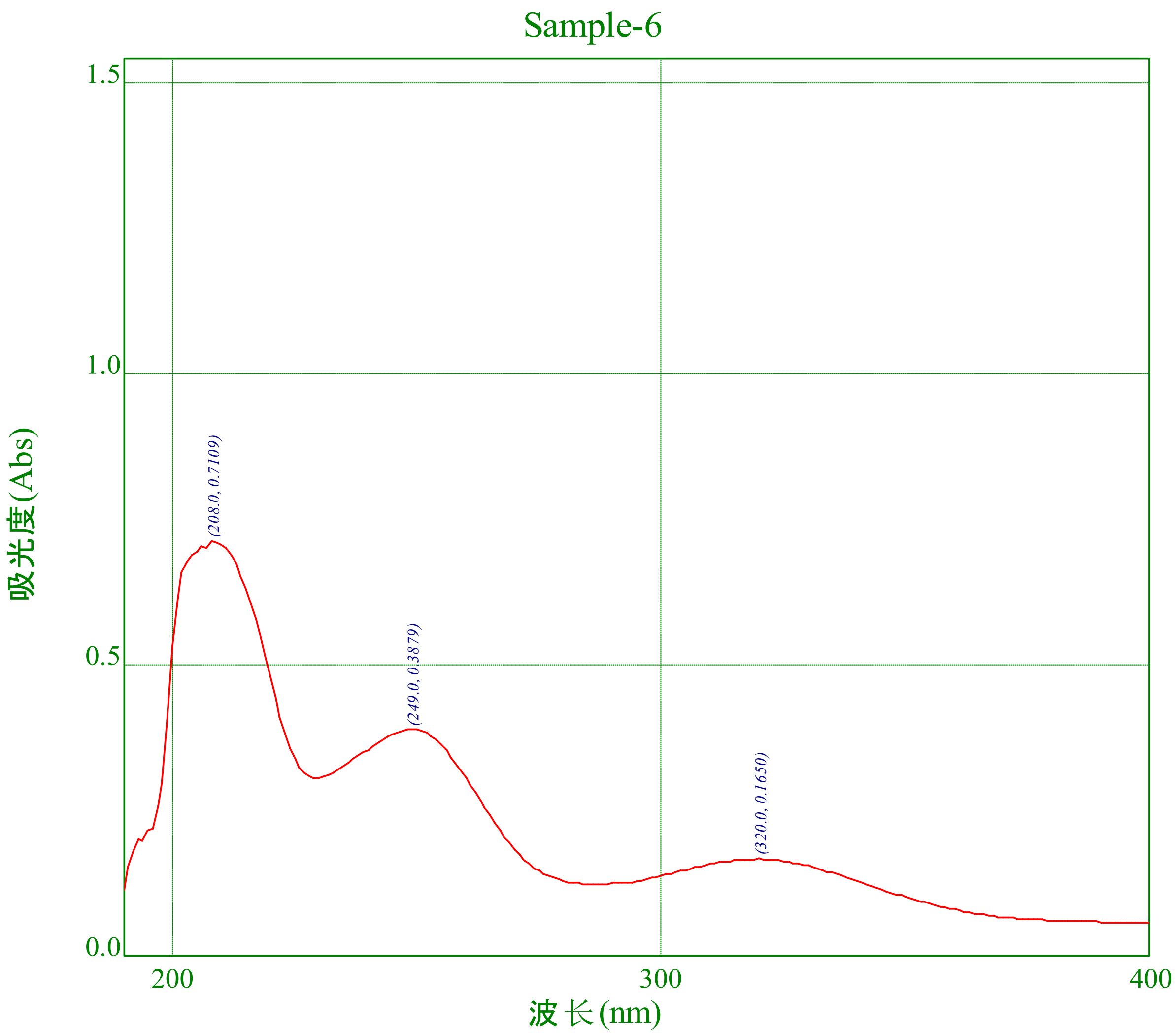

S33 IR spectrum of eutypellenoid C (**3**).

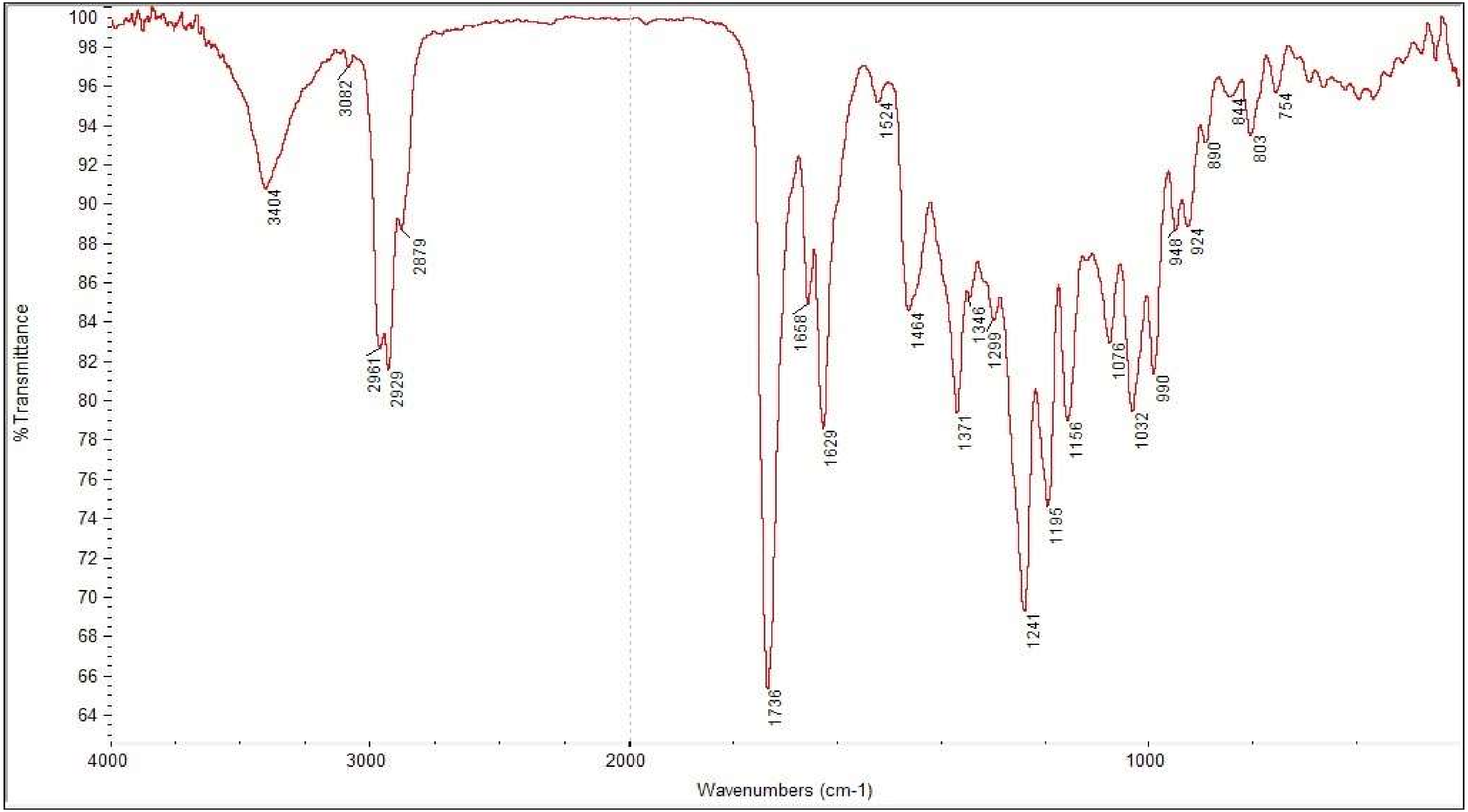

Supplement: Supplementary file 1 [file marinedrugs-16-00284-s001.pdf]
